# DNA methylation markers for kidney function and progression of diabetic kidney disease

**DOI:** 10.1038/s41467-023-37837-7

**Published:** 2023-05-15

**Authors:** Kelly Yichen Li, Claudia Ha Ting Tam, Hongbo Liu, Samantha Day, Cadmon King Poo Lim, Wing Yee So, Chuiguo Huang, Guozhi Jiang, Mai Shi, Heung Man Lee, TRANSCEND Consortium, Hui-yao Lan, Cheuk-Chun Szeto, Robert L. Hanson, Robert G. Nelson, Katalin Susztak, Juliana C. N. Chan, Kevin Y. Yip, Ronald C. W. Ma

**Affiliations:** 1 Department of Computer Science and Engineering, The Chinese University of Hong Kong, Shatin, New Territories Hong Kong; 2 Sanford Burnham Prebys Medical Discovery Institute, La Jolla, CA USA; 3 Department of Medicine and Therapeutics, The Chinese University of Hong Kong, Shatin, New Territories Hong Kong; 4 Hong Kong Institute of Diabetes and Obesity, The Chinese University of Hong Kong, Shatin, New Territories Hong Kong; 5 Laboratory for Molecular Epidemiology in Diabetes, Li Ka Shing Institute of Health Sciences, The Chinese University of Hong Kong, Shatin, New Territories Hong Kong; 6 Department of Medicine, Renal Electrolyte and Hypertension Division, University of Pennsylvania, Philadelphia, PA USA; 7 Institute of Diabetes Obesity and Metabolism, University of Pennsylvania, Philadelphia, PA USA; 8 Phoenix Epidemiology and Clinical Research Branch, National Institute of Diabetes and Digestive and Kidney Diseases, Phoenix, AZ USA; 9 Department of Biochemistry and Molecular Genetics, College of Graduate Studies and Arizona College of Osteopathic Medicine, Midwestern University, Glendale, AZ USA; 10 School of Public Health (Shenzhen), Sun Yat-sen University, Shenzhen, Guangdong China; 11 Li Ka Shing Institute of Health Sciences, The Chinese University of Hong Kong, Shatin, New Territories Hong Kong; 12 Hong Kong Bioinformatics Centre, The Chinese University of Hong Kong, Shatin, New Territories Hong Kong

**Keywords:** Diabetes complications, Translational research, Epigenomics, Kidney diseases

## Abstract

Epigenetic markers are potential biomarkers for diabetes and related complications. Using a prospective cohort from the Hong Kong Diabetes Register, we perform two independent epigenome-wide association studies to identify methylation markers associated with baseline estimated glomerular filtration rate (eGFR) and subsequent decline in kidney function (eGFR slope), respectively, in 1,271 type 2 diabetes subjects. Here we show 40 (30 previously unidentified) and eight (all previously unidentified) CpG sites individually reach epigenome-wide significance for baseline eGFR and eGFR slope, respectively. We also develop a multisite analysis method, which selects 64 and 37 CpG sites for baseline eGFR and eGFR slope, respectively. These models are validated in an independent cohort of Native Americans with type 2 diabetes. Our identified CpG sites are near genes enriched for functional roles in kidney diseases, and some show association with renal damage. This study highlights the potential of methylation markers in risk stratification of kidney disease among type 2 diabetes individuals.

## Abstract (web summary)

Epigenetic markers are potential biomarkers for diabetes and related complications. Here, the authors identify CpG sites associated with kidney function and its subsequent decline using both single-site and multisite analyses, which are shown to have functional significance in the kidney.

## Introduction

There is a global epidemic of type 2 diabetes. The increasing prevalence of young-onset diabetes has contributed to the increasing burden of end-stage kidney disease (ESKD) due to the associated long disease duration^1,2^. Given the preventable nature of diabetic kidney disease (DKD), there is a need to identify individuals at risk of progression of DKD and ESKD for early intensive interventions. Several treatments have recently been proven to retard the progression of DKD, including sodium glucose transporter 2 (SGLT2) inhibitors^3^ and selective mineralocorticoid receptor antagonist such as Finerenone^4^. These expanding treatment options for DKD have increased the urgency to develop new models that can stratify those at high risk of kidney dysfunction.

There have been numerous efforts to identify biomarkers that can guide the stratification of DKD, including the use of genetic and other types of biomarkers. Whilst genome-wide association studies (GWAS) have had considerable success in identifying genetic markers for type 2 diabetes and other complex diseases, the progress in identifying loci associated with DKD had been less impressive^5,6^. Epigenetic markers, including methylation changes and miRNA, may be able to capture the interaction between environmental factors and the genome, and may provide new biomarkers for diabetes-related complications^7^. Methylation markers, in particular, have been postulated to mediate the effects of metabolic memory^8^, and are promising biomarkers for diabetic complications. Some previous studies have investigated DNA methylation changes associated with DKD based on human blood^9,10^, human kidney tubules^11,12^, or mouse samples^13^. These studies involved control samples from healthy individuals or individuals with diabetes.

In this study, we examine whether methylation at CpG sites, measured in peripheral blood, may be associated with renal function, and whether this information can be used to predict deterioration in kidney function in type 2 diabetes for prognostication purpose.

## Results

### Genome-wide DNA methylation trends are associated with baseline kidney function

We studied a cohort of 1271 patients with type 2 diabetes from the Hong Kong Diabetes Register (HKDR). Among the patients, 19.7% had DKD at baseline, defined as eGFR<60 ml/min/1.73 m^2^ (Supplementary Table 1 and Supplementary Fig. 1). During a median follow-up period of 14.6 (Q1–Q3: 8.3–19.4) years, 33% developed ESKD. During the follow-up period, the included subjects had a median number of eGFR measurements of 29 (Q1–Q3: 15-46), and the median eGFR slope during follow-up was −2.27% (Q1–Q3: −9.11 to −0.65) change of eGFR per year.

We profiled the DNA methylome of whole-blood samples of the patients using the Illumina Infinium HumanMethylation450K BeadChip (“Methods”). For the DNA methylation data produced, after filtering and normalization, 434,908 CpG sites and 1268 samples were retained. Data reproducibility was confirmed by replicate samples (Supplementary Results and Supplementary Fig. 2). The top principal components (PCs) of our DNA methylation data were strongly indicative of sex, age, and smoking status (Supplementary Methods, Supplementary Results, and Supplementary Fig. 3), which are consistent with previous studies^14–19^ and further confirm the quality of our data.

DNA methylation was associated with renal function, with the models for baseline eGFR achieving a high mean area under the receiver–operator characteristic (AUROC) of 0.76 (Supplementary Fig. 4a). This association was not due to confounding factors caused by sex, age, or smoking status (Supplementary Results and Supplementary Fig. 4b, c). In contrast, most of the other clinical variables were not strongly associated with DNA methylation (Supplementary Fig. 5).

### Methylation levels of individual CpG sites are associated with baseline renal function and renal function decline

To discover individual CpG sites associated with kidney function, we performed an epigenome-wide association study (EWAS) of baseline eGFR. Since recent studies have reported that CpG methylation levels are predictive of the decline of eGFR over time^11,20^, we also set eGFR slope as an additional target trait. We included sex, age, smoking status, duration of diabetes, hemoglobin A1c, blood pressure, batch of experiment, and cell-type composition estimations^21^ as covariates.

**Fig. 1:**
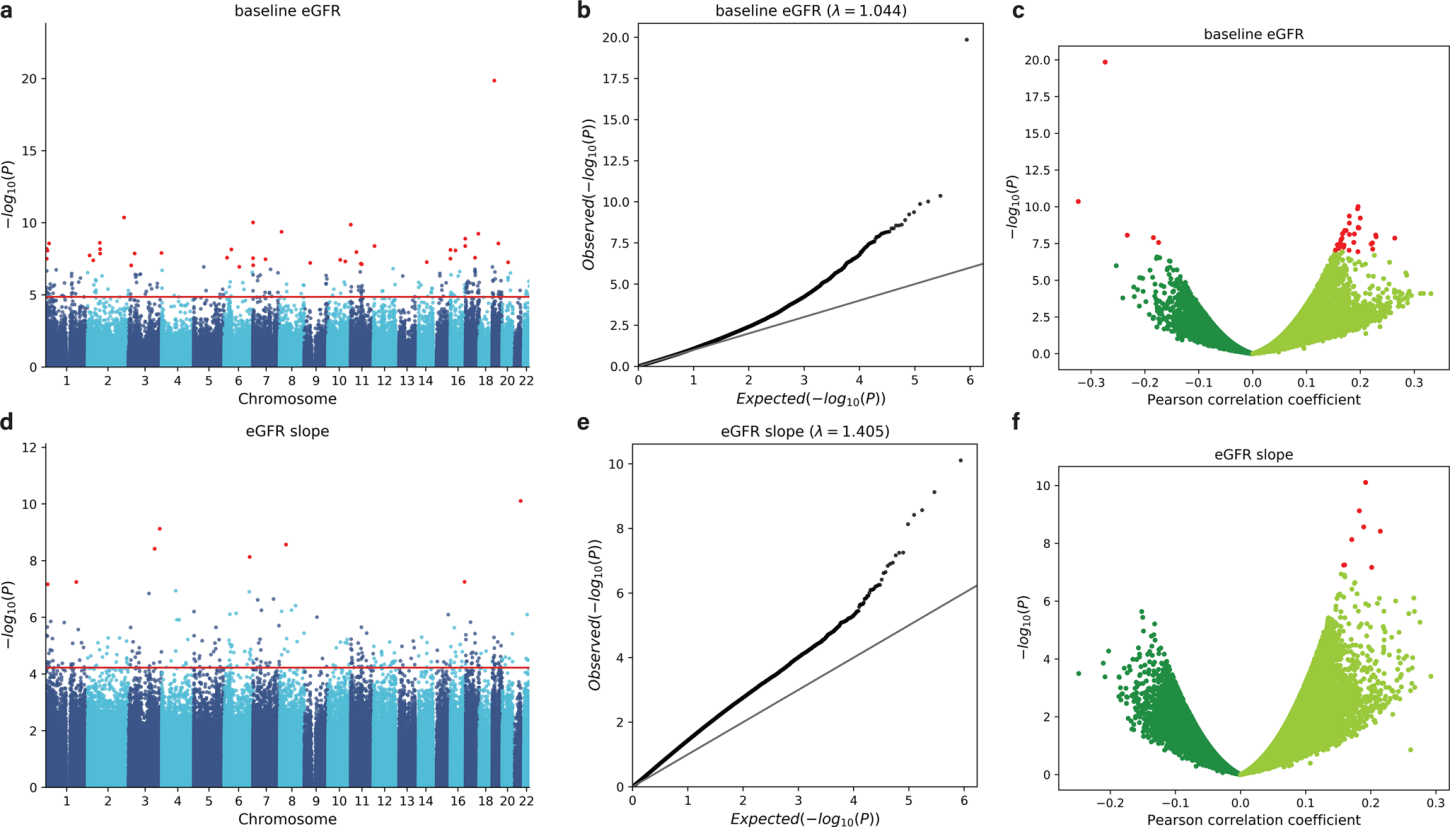
Association between CpG methylation and renal function. The methylation level of each CpG site was tested for its association with baseline eGFR (**a**–**c**) and eGFR slope (**d**–**f**). The results of all the 434,908 CpG sites analyzed in this study are shown using Manhattan plots (**a**, **d**), quantile–quantile (QQ) plots (**b**, **e**), and volcano plots (**c**, **f**). *P* values were computed using two-sided Student’s *t* test. In the Manhattan plots, CpG sites with a Bonferroni-corrected *P* value <0.05 are shown in red. The horizontal red lines show the cutoff above which all sites are significant at FDR = 0.05. In the QQ plots, the diagonal straight line is the expectation under the null hypothesis. λ is the inflation factor. In the volcano plots, CpG sites with a Bonferroni-corrected *P* value <0.05 are shown in red.

For baseline eGFR, 40 CpG sites reached epigenome-wide significance (Bonferroni-corrected *P* value below 0.05) and 386 CpG sites were statistically significant at FDR = 0.05 (Fig. 1a–c, Table 1, and Supplementary Data 1). The most significant CpG site was cg17944885 (Bonferroni-corrected *P* = 6.11 × 10^−15^), located between *ZNF788* and *ZNF20* on chromosome 19. The DNA methylation level of this CpG site had also been associated with kidney function in various populations^22–25^ (Supplementary Fig. 6 and Supplementary Data 1 and 2). Interestingly, two of the sites with a Bonferroni-corrected*P* value below 0.05 (cg04983687, cg01676795) and one other significant site at FDR = 0.05 (cg22460173) in our cohort had also been reported as significant in a recent multiethnic meta-analysis^22^, but they had not been reported to have a significant association with kidney function in earlier studies of cohorts that involved a single ethnic group^10,22^.

**Table 1:** CpG sites with their methylation levels significantly associated with baseline eGFR or eGFR slope in the single-site analysis

CpG site	Genomic location	Model coefficient	*P* value	Corrected *P* value	Annotated gene(s)	Gene region(s)
**Baseline eGFR**						
cg17944885	Chr19:12,225,735	−5.156	1.41E-20	6.11E-15	–	–
cg25364972	Chr2:217,075,573	−6.303	4.36E-11	1.90E-05	–	–
cg06449934	Chr7:1,130,697	3.679	9.70E-11	4.22E-05	*GPER*	5’ UTR
					*C7orf50*	Gene body
cg02304370	Chr11:587,926	3.662	1.37E-10	5.97E-05	*PHRF1*	Gene body
cg21919729	Chr8:11,719,367	3.368	4.28E-10	1.86E-04	*CTSB*	5’ UTR
cg04610187	Chr17:76,360,794	3.766	5.83E-10	2.53E-04	–	–
cg04983687	Chr16:88,558,223	3.372	1.29E-09	5.61E-04	*ZFPM1*	Gene body
cg27254661	Chr2:73,118,624	3.697	2.47E-09	0.001	SPR	Gene body
cg18593194	Chr19:36,205,201	3.697	2.75E-09	0.001	*ZBTB32*	5’ UTR
cg12065228	Chr1:19,652,788	3.721	2.76E-09	0.001	*PQLC2*	Gene body
cg08940169	Chr16:88,540,241	3.260	4.16E-09	0.002	*ZFPM1*	Gene body
cg19434937	Chr12:7,104,184	3.206	4.16E-09	0.002	*LPCAT3*	Gene body
cg11699125	Chr1:6,341,327	3.144	6.55E-09	0.003	*ACOT7*	Gene body
cg17988187	Chr2:74,612,222	3.131	6.84E-09	0.003	*LOC100189589*	TSS1500
cg09823543	Chr6:43,146,056	3.557	7.10E-09	0.003	*SRF*	Gene body
cg02475695	Chr16:616,220	3.378	7.63E-09	0.003	*NHLRC4*	TSS1500
cg06972908	Chr16:30,488,321	4.344	8.35E-09	0.004	*ITGAL*	Gene body
cg11544657	Chr1:9,968,130	−4.430	8.61E-09	0.004	*CTNNBIP1*	5’ UTR
cg23845009	Chr11:34,323,678	4.360	1.09E-08	0.005	*ABTB2*	Gene body
cg09610644	Chr3:197,249,274	−3.469	1.26E-08	0.005	*BDH1*	Gene body
cg12981272	Chr3:37,281,848	5.063	1.36E-08	0.006	–	–
cg12077754	Chr2:75,089,669	3.114	1.38E-08	0.006	*HK2*	Gene body
cg10142874	Chr2:11,917,623	3.074	1.86E-08	0.008	*LPIN1*	Gene body
cg00934987	Chr17:56,605,468	3.540	2.68E-08	0.012	*SEPT4*	Gene body
cg22753611	Chr6:17,472,892	−3.284	2.68E-08	0.012	*CAP2*	Gene body
cg04816311	Chr7:1,066,650	4.226	2.88E-08	0.013	*C7orf50*	Gene body
cg04497992	Chr16:616,212	3.053	3.11E-08	0.014	*NHLRC4*	TSS1500
cg09249800	Chr1:6,341,287	3.042	3.15E-08	0.014	*ACOT7*	Gene body
cg01676795	Chr7:75,586,348	4.178	3.43E-08	0.015	*POR*	Gene body
cg25854298	Chr10:73,936,754	2.952	3.79E-08	0.016	*ASCC1*	Gene body
cg10489463	Chr2:33,546,572	3.190	4.07E-08	0.018	*LTBP1*	Gene body
cg23516680	Chr10:103,923,333	3.105	4.89E-08	0.021	*NOLC1*	3’ UTR
cg02170785	Chr14:69,650,830	3.012	5.44E-08	0.024	–	–
cg19448292	Chr20:35,504,064	3.177	5.59E-08	0.024	*C20orf118*	TSS1500
cg01499988	Chr9:35,755,346	2.980	6.16E-08	0.027	*MSMP*	TSS1500
cg25087851	Chr11:60,623,918	2.993	6.95E-08	0.030	*GPR44*	TSS1500
cg22406869	Chr11:66,276,941	4.239	7.63E-08	0.033	*DPP3*	3’ UTR
					*BBS1*	TSS1500
cg18650626	Chr7:1,914,073	2.886	8.89E-08	0.039	*MAD1L1*	Gene body
cg00506299	Chr3:16,469,127	3.373	9.14E-08	0.040	*RFTN1*	Gene body
cg16809457	Chr6:90,399,677	3.694	1.14E-07	0.050	*MDN1*	Gene body
**eGFR slope**						
cg10272901	Chr21:46,677,879	1.316	7.84E-11	3.41E-05	–	–
cg12354056	Chr3:186,136,503	1.126	7.50E-10	3.26E-04	–	–
cg18461548	Chr8:37,701,921	1.179	2.72E-09	0.001	*BRF2*	3’ UTR
cg00695821	Chr3:156,124,891	1.354	3.81E-09	0.002	*KCNAB1*	Gene body
cg22822893	Chr6:15,1662,789	1.056	7.39E-09	0.003	*AKAP12*	Gene body
cg02566611	Chr16:83,948,975	0.986	5.61E-08	0.024	*MLYCD*	Gene body
cg20741134	Chr1:181,382,639	0.976	5.67E-08	0.025	–	–
cg04027328	Chr1:11,372,138	1.290	6.81E-08	0.030	–	–

*P* values were computed using two-sided Student’s *t* test. Each listed site has a Bonferroni-corrected *P* value <0.05. TSS1500: the region between 200 bp and 1500 bp upstream of the transcription start site (TSS). In the model coefficients, a positive sign means that a higher methylation level is associated with higher baseline eGFR or slower eGFR decline, while a negative sign means the opposite.

For eGFR slope, eight CpG sites had a Bonferroni-corrected*P* value below 0.05 and 74 CpG sites were significant at FDR = 0.05 (Fig. 1d–f, Table 1, and Supplementary Data 1). The most significant CpG site was cg10272901 (Bonferroni-corrected *P* = 3.41 × 10^−5^), located in a CpG island on chromosome 21. None of these 74 sites was reported to be associated with eGFR slope in previous studies, conducted mainly in the general population rather than population with diabetes (Supplementary Data 1 and  2). When we performed reciprocal lookup of the previously reported top sites, we found several sites reported by Gluck et al., based on data from multiple populations^11^, to have marginally significant *P* values in our data (Supplementary Fig. 6). These included cg15826891 (*P* = 5.29 × 10^−5^ in our data), which is located within the *MIR100HG*non-coding gene locus on chromosome 11, and cg02950701 (*P* = 1.26 × 10^−4^ in our data), which is located within the protein-coding gene *CCNY* locus on chromosome 10.

### A multisite approach to identifying sets of CpG sites indicative of renal function

The single-site approach described above, though commonly used in the literature, has two important limitations. First, some CpG sites that are not strongly associated with kidney function by themselves could complement other sites to explain residual kidney function differences. These “auxiliary” sites cannot be identified by the single-site approach. Second, some significant CpG sites identified by the single-site approach could be strongly correlated with each other (Supplementary Fig. 7), due to genomic spatial dependency or other reasons, leading to redundancy and diversion of attention to non-functional sites.

To tackle these limitations, we developed a multisite approach that considered all CpG sites at the same time and selected a subset of them to create the best model to infer baseline eGFR or eGFR slope (“Methods”). Considering both the model performance and complexity of the models, our procedure automatically determined the feature selection thresholds (“Methods” and Supplementary Results). According to left-out testing data not involved in this procedure, at these selected thresholds, the Pearson correlation between the measured baseline eGFR values and the values inferred by the models was 0.704, and that of eGFR slope was 0.386 (Supplementary Fig. 8a, d).

### The multisite models capture relationships between DNA methylation and renal function in multiple populations

After confirming the validity of our procedure, we then implemented it to rebuild the models using the whole set of samples. In these “final” models, 64 and 37 CpG sites were included for predicting baseline eGFR and eGFR slope, respectively (Tables 2 and  3 and Supplementary Data 3).

**Table 2:** CpG sites in the final multisite model for baseline eGFR

CpG site	Genomic location	Model coefficient		Single-site corrected *P* value	Annotated gene(s)	Gene region(s)
		With covariates	Without covariates			
cg17944885	Chr19:12225735	−3.291	−4.211	6.11E-15	–	–
cg06449934	Chr7:1130697	0.442	0.088	4.22E-05	*GPER*	5’ UTR
					*C7orf50*	Gene body
cg02304370	Chr11:587926	0.491	0.313	5.97E-05	*PHRF1*	Gene body
cg21919729	Chr8:11719367	0.778	0.715	1.86E-04	*CTSB*	5’ UTR
cg04610187	Chr17:76360794	0.656	0.721	2.54E-04	–	–
cg18593194	Chr19:36205201	1.661	1.188	0.001	*ZBTB32*	5’ UTR
cg12065228	Chr1:19652788	0	0	0.001	*PQLC2*	Gene body
cg09823543	Chr6:43146056	1.127	1.047	0.003	*SRF*	Gene body
cg23845009	Chr11:34323678	2.249	1.145	0.005	*ABTB2*	Gene body
cg09610644	Chr3:197249274	−1.780	−2.809	0.005	*BDH1*	Gene body
cg00934987	Chr17:56605468	0	0.661	0.012	*SEPT4*	Gene body
cg04497992	Chr16:616212	0.116	0	0.014	*NHLRC4*	TSS1500
cg01676795	Chr7:75586348	1.939	1.225	0.015	*POR*	Gene body
cg00506299	Chr3:16469127	1.464	0.713	0.040	*RFTN1*	Gene body
cg01885635	Chr3:40566085	1.877	3.159	0.169	*ZNF621*	TSS1500
cg15232319	Chr19:4376459	0	−0.557	0.414	*SH3GL1*	Gene body
cg20062057	Chr2:50201479	1.508	1.428	0.466	*NRXN1*	Gene body
cg07397612	Chr22:47423986	1.452	1.613	0.497	*TBC1D22A*	Gene body
cg20970369	Chr1:111744108	−1.123	−1.395	0.658	*DENND2D*	TSS1500
cg13091627	Chr1:153518476	−1.825	−1.504	0.851	*S100A4*	TSS200
cg23511909	Chr3:128340787	0.555	0.722	0.887	*RPN1*	Gene body
cg02835823	Chr16:85979060	−0.451	0	0.902	–	–
cg20133890	Chr6:31680144	0	0	1	*LY6G6E*	Gene body
cg12465678	Chr1:27953336	0.045	−1.188	1	*FGR*	TSS1500
cg20299697	Chr3:138069423	0.764	1.401	1	*MRAS*	5’ UTR
cg14141741	Chr7:947428	1.157	0.893	1	*ADAP1*	Gene body
cg19458497	Chr11:63403371	0.848	0.972	1	*ATL3*	Gene body
cg10578938	Chr5:156695410	−0.565	−0.667	1	*CYFIP2*	5’ UTR
cg22049753	Chr2:240895815	1.292	1.216	1	–	–
cg26344619	Chr14:76046018	1.082	0.987	1	*FLVCR2*	Gene body
cg11845111	Chr2:191398756	−1.155	−1.506	1	*TMEM194B*	Gene body
cg23509869	Chr6:31553441	−1.424	−0.488	1	*LST1*	TSS1500
cg14583999	Chr3:10019040	0.691	1.162	1	*TMEM111*	Gene body
cg06943835	Chr11:64662577	0.734	1.908	1	*ATG2A*	Gene body
cg19597449	Chr19:8117924	0.909	0	1	*CCL25*	TSS200
cg26336935	Chr17:39769213	1.045	1.218	1	*KRT16*	TSS200
cg23261820	Chr5:102382738	1.311	1.636	1	–	–
cg07781445	Chr17:2886250	0	0.727	1	*RAP1GAP2*	Gene body
cg18036734	Chr5:177036766	0.495	0	1	*B4GALT7*	3’ UTR
cg01924561	Chr1:43416103	−1.267	−1.538	1	*SLC2A1*	Gene body
cg07477034	Chr17:53341969	1.128	1.754	1	*HLF*	TSS1500
cg24707889	Chr21:46341304	−0.252	0.217	1	*ITGB2*	5’UTR
cg00501876	Chr3:39193251	−2.161	−1.533	1	*CSRNP1*	5’UTR
cg25013303	Chr1:10961257	0.042	0.387	1	–	–
cg18070458	Chr11:121319927	−0.802	−0.611	1	–	–
cg11961845	Chr7:129008179	−0.606	−0.081	1	*AHCYL2*	Gene body
cg17124293	Chr10:45403981	−1.490	−1.360	1	–	–
cg13408344	Chr15:31631240	−0.665	−0.627	1	*KLF13*	Gene body
cg19893929	Chr2:16105823	−0.103	0	1	–	–
cg00791074	Chr6:151186169	0	0.079	1	*MTHFD1L*	TSS1500
cg26608718	Chr19:15530737	0.238	1.443	1	*AKAP8L*	TSS1500
cg01955153	Chr16:50769852	−0.380	0	1	–	–
cg06015525	Chr12:57872123	−1.678	−1.772	1	*ARHGAP9*	Gene body
cg16324121	Chr3:9954273	0	−1.235	1	*IL17RE*	Gene body
cg05062653	Chr5:562341	−1.604	−1.597	1	–	–
cg03881294	Chr2:11884333	0	0	1	–	–
cg12171761	Chr8:61910949	−0.200	−0.349	1	–	–
cg00912580	Chr2:135169533	−0.107	−0.145	1	*MGAT5*	Gene body
cg26687842	Chr13:41055491	−1.335	−1.991	1	*LOC646982*	TSS1500
cg27376617	Chr7:30518048	1.132	1.501	1	*NOD1*	5’ UTR
cg03032497	Chr14:61108227	0	−1.895	1	–	–
cg09511896	Chr1:228246937	−1.370	−1.690	1	*WNT3A*	Gene body
cg03607117	Chr3:53080440	−1.360	−3.570	1	*SFMBT1*	TSS1500
cg18473521	Chr12:54448265	−0.651	−1.655	1	*HOXC4*	Gene body

Sites with a zero coefficient in a model are those that were originally selected by our procedure as input for the LASSO method to consider but were finally not given a nonzero weight. TSS200: the region between the transcription start site (TSS) and 200 bp upstream of it. TSS1500: the region between 200 bp and 1500 bp upstream of the TSS. In the model coefficients, a positive sign means that a higher methylation level is associated with higher baseline eGFR or slower eGFR decline, while a negative sign means the opposite. Single-site corrected *P* value: Bonferroni-corrected *P* values in the EWAS results.

**Table 3:** CpG sites in the final multisite model for eGFR slope

CpG site	Genomic location	Model coefficient		Single-site corrected *P* value	Annotated gene(s)	Gene region(s)
		With covariates	Without covariates			
cg10272901	Chr21:46677879	0.684	0.679	3.41E-05	–	–
cg12354056	Chr3:186136503	0.255	0.345	3.26E-04	–	–
cg22822893	Chr6:151662789	0.075	0.035	0.003	*AKAP12*	Gene body
cg04027328	Chr1:11372138	0.243	0.005	0.030	–	–
cg16425726	Chr4:83680145	0.403	0.385	0.050	*SCD5*	Gene body
cg21368479	Chr6:149415018	0.702	0.683	0.055	–	–
cg22930808	Chr3:122281881	0.386	0.352	0.063	*PARP9*	5’ UTR
					*DTX3L*	TSS1500
cg01647632	Chr15:89438905	0.477	0.476	0.350	*HAPLN3*	TSS200
cg13591783	Chr9:75768868	0.598	0.625	0.429	*ANXA1*	5’ UTR
cg10761425	Chr3:12988976	−0.575	−0.517	0.991	*IQSEC1*	Gene body
cg15989436	Chr5:150465875	0.110	0	1	–	–
cg23047271	Chr3:64210991	0.476	0.615	1	*PRICKLE2*	First exon
cg02647990	Chr3:196230837	0.612	0.553	1	*RNF168*	TSS1500
cg05580141	Chr12:49071788	0	−0.153	1	*C12orf41*	Gene body
cg17944885	Chr19:12225735	−0.758	−1.061	1	–	–
cg04383715	Chr16:34209247	0.662	0.653	1	–	–
cg14943908	Chr6:31589196	0	−0.049	1	*BAT2*	5’ UTR
cg07723558	Chr17:7184224	0.383	0.456	1	*SLC2A4*	TSS1500
cg06575692	Chr16:68112968	−0.494	−0.615	1	*DUS2L*	3’ UTR
cg11494773	Chr7:48128242	0	0.197	1	*UPP1*	TSS200
cg16933224	Chr11:63604740	0.141	0.336	1	–	–
cg25686812	Chr3:42597657	−0.286	−0.298	1	*SEC22C*	Gene body
cg04697209	Chr16:20087376	−0.538	−0.627	1	–	–
cg12526474	Chr7:140097579	0.147	0.314	1	*SLC37A3*	5’ UTR
cg06681597	Chr17:13972703	−0.611	−0.725	1	*COX10*	TSS200
cg20010135	Chr16:30996822	0	0.084	1	*HSD3B7*	5’ UTR
cg20101066	Chr7:148581385	−0.607	−0.690	1	*EZH2*	5’ UTR
cg08626625	Chr6:33129765	0.107	−0.034	1	–	–
cg21926091	Chr8:141108607	−0.031	−0.300	1	*TRAPPC9*	Gene body
cg15581429	Chr19:39369353	−0.648	−0.458	1	*SIRT2*	3’ UTR
					*RINL*	TSS1500
cg19693031	Chr1:145441552	0.931	1.428	1	*TXNIP*	3’ UTR
cg21693780	Chr2:15731793	0	0.109	1	*DDX1*	First exon
cg10639435	Chr8:146104221	−0.143	−0.383	1	*ZNF250*	3’ UTR
cg12245040	Chr16:2009320	0.019	0.145	1	*NDUFB10*	TSS200
cg05166473	Chr16:88103629	−0.371	−0.293	1	*BANP*	Gene body
cg20728490	Chr10:98064175	−0.145	−0.090	1	*DNTT*	5’ UTR
cg22293458	Chr3:184483865	−0.550	−0.493	1	–	–

Sites with a zero coefficient in a model are those that were originally selected by our procedure as input for the LASSO method to consider but were finally not given a nonzero weight. TSS200: the region between the transcription start site (TSS) and 200 bp upstream of it. TSS1500: the region between 200 bp and 1500 bp upstream of the TSS. In the model coefficients, a positive sign means that a higher methylation level is associated with higher baseline eGFR or slower eGFR decline, while a negative sign means the opposite. Single-site corrected *P* value: Bonferroni-corrected *P* values in the EWAS results.

**Fig. 2:**
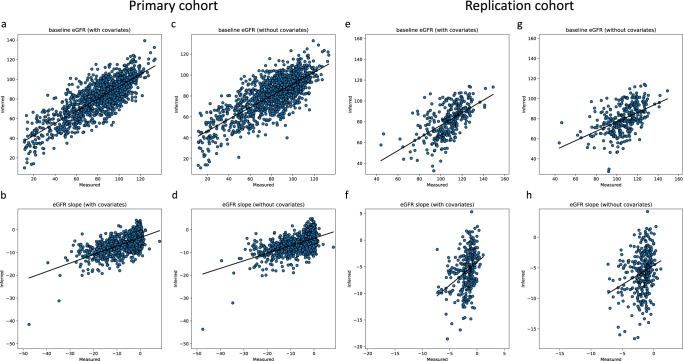
Performance of the multisite models. Scatter plots of inferred baseline eGFR and eGFR slope against their corresponding actual measurements using selected CpG sites based on the models constructed from the primary cohort and applied to the primary cohort (**a**–**d**) or the Native American cohort (trained using CpG sites available to both cohorts) (**e**–**h**). In each panel, the black lines mark the best fit lines of linear regression. Source data are provided as a Source Data file.

For baseline eGFR and eGFR slope, the actual values and the values inferred by our final models had Pearson correlations of 0.806 and 0.635, respectively (Table 4 and Fig. 2a, b). The performance of the models was better with the covariates than without (Table 4 and Fig. 2c, d), and they were substantially better than models constructed from the same number of random CpG sites (Supplementary Fig. 9) and several alternative models (Supplementary Results).

**Table 4:** Performance of the multisite models constructed from data of the primary cohort and applied to either the primary or Native American cohort (trained using CpG sites available to both cohorts)

Testing cohort	Target phenotype	CpG sites	Covariates	PCC	SCC	MAE
Primary	Baseline eGFR	64	Yes	0.806	0.762	11.707
			No	0.765	0.717	12.815
	eGFR slope	37	Yes	0.635	0.584	4.119
			No	0.589	0.532	4.327
Primary (only CpG sites common to both cohorts)	Baseline eGFR	59	Yes	0.801	0.759	11.838
			No	0.759	0.712	12.957
	eGFR slope	29	Yes	0.612	0.564	4.202
			No	0.562	0.507	4.430
Native Americans	Baseline eGFR	59	Yes	0.591	0.614	26.947
			No	0.497	0.534	27.528
	eGFR slope	29	Yes	0.356	0.389	4.260
			No	0.273	0.279	4.274

*PCC* Pearson correlation coefficient, *SCC* Spearman correlation coefficient, *MAE* mean absolute error.

The “CpG sites” column shows the number of sites selected by our procedure as input for the LASSO method to consider, some of which finally got assigned a zero weight by LASSO.

In our final models, some of the CpG sites included were also significantly associated with kidney function in the single-site analysis, such as the most significant sites cg17944885 for baseline eGFR and cg10272901 for eGFR slope. Other sites demonstrated significant associations only in the multisite models, showing that they carried additional information for inferring the target traits. Intriguingly, the most significant site cg17944885 for baseline eGFR was also included in the multisite model for eGFR slope, although it was not significant for eGFR slope in the single-site analysis. One of the selected sites for the baseline eGFR model, cg13408344, was previously associated with baseline eGFR^22^.

To evaluate whether the selected sites could successfully classify people with or without kidney disease, we constructed regularized logistic regression models using the above choices of CpG sites for baseline eGFR and eGFR slope. All the models performed well in these classification tasks, achieving a mean AUROC of 0.89 for baseline eGFR and 0.81 for eGFR slope (Supplementary Table 2), demonstrating the ability of these sites to recognize people with potential renal dysfunction.

In the final models, since all samples were used in training the model, there were no left-out samples for evaluating the model performance in an unbiased fashion. Therefore, we further tested our models using genome-wide methylation measurements of blood samples from an independent cohort of 326 Native American subjects with type 2 diabetes. The results (Table 4, Supplementary Table 3, Fig. 2e–h, and Supplementary Fig. 10) show that our models also achieved good performance for predicting baseline eGFR and eGFR decline in type 2 diabetes in this independent cohort despite differences in ethnicity.

### Proximal genes of the selected sites in the single-site and multisite analyses have potential kidney functions

We next evaluated the functional significance of the genes proximal to (within 1 kb) the sites identified in our single-site and multisite analyses by checking whether they have been reported as potentially related to kidney function. We collected these potential kidney function-related genes from a number of previous studies that identified the genes using various types of data, including DNA methylation data of blood samples from people with or without kidney disease^9,26–28^, bulk RNA expression data of human kidneys^29–31^, single-cell RNA sequencing data of mouse kidneys^32,33^, and GWAS prioritized genes^34,35^.

**Fig. 3:**
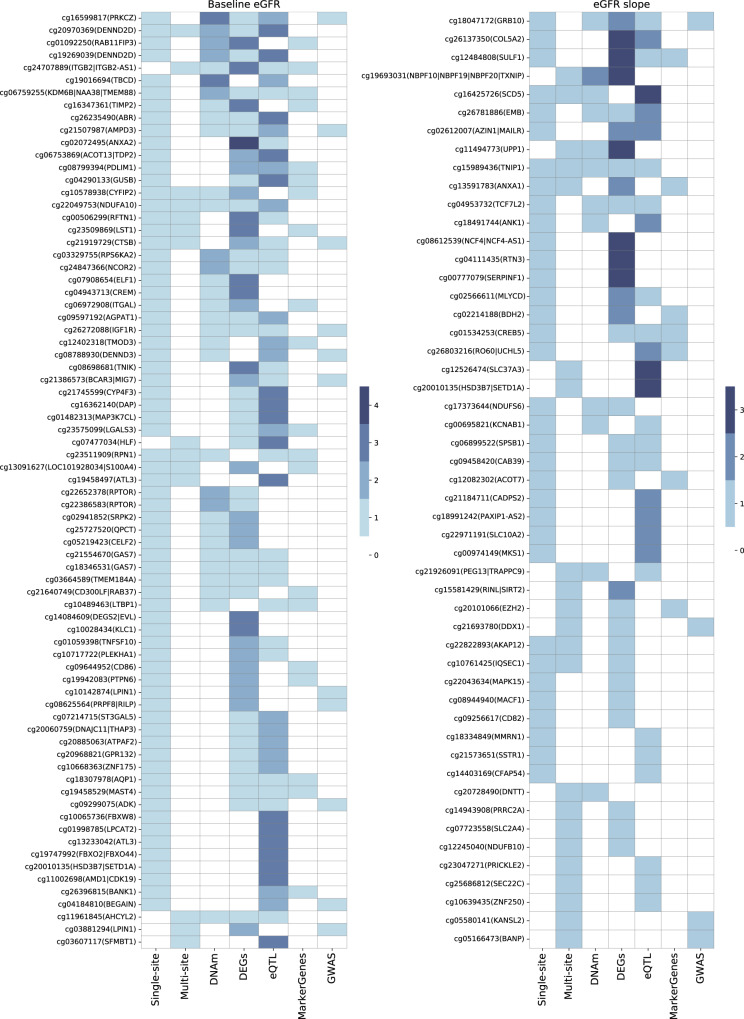
Support for the functional significance of genes near the CpG sites identified in our single-site and multisite analyses. Each row corresponds to a CpG site and all genes within 1 kb from it. The “Single-site” and “Multi-site” columns show whether a site is significant at FDR = 0·05 in our single-site analysis and whether it is included in the final multisite model, respectively. The “DNAm” and “DEGs” columns show whether at least one of the nearby genes is differentially methylated or differentially expressed in samples with and without kidney function decline in one or more previous methylation^9,25–28^ or gene expression studies^29,31,32^, respectively. The “eQTL” column shows whether at least one of the nearby genes is associated with an expression quantitative trait locus identified in human kidney samples in a previous study^30^. The “MarkerGenes” column shows whether at least one of the nearby genes is a cell-type-specific marker of a major kidney cell type as identified previously^33^. The “GWAS” column shows whether at least one of the nearby genes is prioritized by GWAS results in two recent studies^34,35^. Only CpG sites where the nearby genes have at least 3 and 1 functional supports, respectively, for baseline eGFR and eGFR slope, are shown.

Of the 348 CpG sites (which corresponded to 358 genes) identified by our single-site and multisite analyses as associated with baseline eGFR and proximal to (within 1 kb) annotated genes, 228 (which corresponded to 215 genes) of them (65.5%) were reported in at least one of these previous studies (Fig. 3 and Supplementary Data 4), which corresponded to a 1.25-fold enrichment as compared to the set of all human genes (*P* = 3.78 × 10^−6^, hypergeometric test).

Noticeably, the CpG site cg24707889, located in the upstream region of the *ITGB2* gene, was identified in the multisite model but not recognized as significant at FDR = 0.05 in the single-site analysis. The association between *ITGB2* and kidney function was supported by various data such as blood DNA methylation^27^, RNA expression and expression quantitative trait loci (eQTLs) in human kidney samples^30,31^, and single-cell RNA expression in mouse kidneys^32,33^. The *ITGB2* gene encodes integrin subunit beta 2 (also known as archetypal innate immune receptor CD11b/CD18), which plays an important role in immune response, and defects in this gene may cause leukocyte adhesion deficiency. A recent study reported that inhibition of CD11b/CD18 prevented long-term fibrotic ESKD from acute kidney injury (AKI) in cynomolgus monkeys^36^.

Interestingly, our analysis identified several previously unidentified CpG sites associated with baseline eGFR with nearby genes having differential expression between samples from people with and without kidney disease, such as *RFTN1* and *CTSB* (“Discussion”).

For eGFR slope, 51 of the 76 CpG sites (67.1%) (which corresponded to 52 of 89 genes) were reported as potentially related to kidney function in previous studies (Fig. 3 and Supplementary Data 4), which corresponded to a 1.21-fold enrichment as compared to the set of all human genes (*P* = 0.03, hypergeometric test).

One CpG site, cg19693031, which was selected by our multisite model but not recognized as significant at FDR = 0.05 in the single-site analysis, is located in the 3’-UTR (untranslated region) of the *TXNIP* gene. *TXNIP* encodes a thioredoxin-interacting protein, which was implicated in the pathogenesis of DKD. CpG sites within this gene were differentially methylated between patients with type 1 diabetes with and without complications^26^. *TXNIP* expression is related to DKD^31^, structural abnormalities such as cortical interstitial fractional volume (VvInt)^29^, an index of tubule-interstitial damage, as well as folic acid nephropathy (FAN)^32^. Previous studies suggested that hyperglycemia might contribute to DKD by increasing the level of inflammatory factors via upregulating the expression of *TXNIP* through histone modifications, such as increase in H3K9ac, H3K4me3, and H3K4me1, and decrease in H3K27me3 at *TXNIP* promoter region, whereas the contributory roles of DNA methylation required further elucidation^37,38^. Another CpG site, cg13591783, identified in both our single-site and multisite analyses for eGFR slope, is located within the *ANXA1* gene. *ANXA1* encodes annexin A1, which is a membrane-localized protein that binds phospholipids, inhibits phospholipase A2, and has anti-inflammatory activity. *ANXA1* was differentially expressed in kidney tubules between human samples of DKD and control samples^31^ and correlated with VvInt^29^ in patients with DKD. In addition, annexin A1 was a potential therapeutic target in diabetes and the treatment of microvascular disease such as DKD^39,40^.

To further confirm that the CpG sites identified in our single-site and multisite analyses are statistically near genes related to kidney function, we sampled many sets of random genes proximal to (within 1 kb) CpG sites profiled by the Infinium HumanMethylation450 BeadChip with the same sizes as the actual numbers of genes proximal to the CpG sites we identified in our analyses. For these random sets of genes, the numbers of them related to kidney function were significantly smaller than our actual sets of genes identified (Supplementary Fig. 11).

Taken together, among the genes near the CpG sites associated with baseline eGFR or eGFR slope in our single-site and multisite analyses, many of them had been reported to be related to normal kidney function or kidney diseases. These results were based on various types of data, including data curated from human kidney samples, which provides strong support for the functional relevance of our reported CpG sites obtained from blood samples.

To further validate the relevance of our selected CpG sites in the kidney, we selected seven CpG sites that were associated with baseline eGFR in our single-site and multisite analyses, namely cg21573651, cg17944885, cg06449934, cg02304370, cg21919729, cg04610187, and cg18593194 (“Methods”). For two of these seven CpG sites (cg21573651 and cg04610187) their methylation levels in kidney samples were significantly different between kidney disease patients and control groups (Supplementary Fig. 12a, d). Their methylation levels in kidney samples also had significant correlations with eGFR and fibrosis (Supplementary Fig. 12b, c, e, f). These results further supported that the CpG sites we identified from blood samples had functional significance in the kidney. In a different cohort of 84 individuals with type 2 diabetes from the Native American population, two out of the seven CpG sites identified (cg02304370 and cg18593194) showed a suggestive association between methylation measured in peripheral blood with global glomerular sclerosis on morphometric variables of kidney biopsy samples in the same individuals (Supplementary Table 4), again highlighting the potential link between methylation level in blood and kidney pathology.

### eGFR slope inferred by the multisite model can predict future renal failure

There are existing risk equations using clinical variables to predict kidney-related outcomes such as ESKD^41–45^. To see if our multisite model for eGFR slope can also predict future ESKD cases, we used it to predict the 5-year eGFR value of each patient and then determined the corresponding 5-year ESKD status based on it (defined as calculated eGFR<15 ml/min/1.73 m^2^). To avoid over-fitting, we inferred the eGFR slope and predicted the ESKD risk using a cross-validation procedure, in which the multisite model was built on the training samples and the inference was made on the left-out validation samples. The performance of predictions was then evaluated by considering all these left-out predictions together. For the benchmarking purpose, we also predicted 5-year ESKD status of all patients using three clinical risk equations. These included the Joint Asia Diabetes Evaluation (JADE) model developed in Chinese patients with type 2 diabetes^41,46^, an equation based on data from the United Kingdom Prospective Diabetes Study (UKPDS)^44^, and a simple equation that ranks patients by the negative values of baseline eGFR where a lower baseline eGFR value predicted a higher risk of developing ESKD in 5 years.

**Fig. 4:**
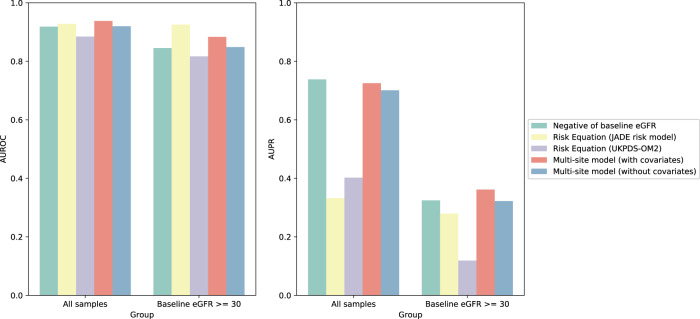
Performance of risk scores by risk equations and the multisite models. AUROC and AUPR of the risk scores from simple negative value of baseline eGFR, JADE risk model, UKPDS-OM2, and our multisite models with or without covariates. The risk scores of the JADE model and UKPDS-OM2 were calculated with the risk equations in the original paper. The risk scores of the multisite models were calculated using the inferred eGFR slope with 5-fold cross-validation. Source data are provided as a Source Data file.

When considering all patients, our multisite model with covariates achieved an AUROC of 0.94 and an AUPR (area under the precision-recall curve) of 0.73 (Fig. 4). When excluding patients with baseline eGFR<30 ml/min/1.73 m^2^, who had very high risk of developing ESKD in 5 years, our model with covariates achieved an AUROC of 0.88 and an AUPR of 0.36 (Fig. 4). In both cases, the performance of our model, even without clinical covariates, was comparable to the performance of the clinical equations, and the inclusion of clinical covariates further enhanced the performance of the models.

In an independent nested case–control cohort of 181 Native Americans with type 2 diabetes, of which 80 developed ESKD during follow-up, baseline methylation scores for baseline eGFR or eGFR slope were both associated with incident ESKD (Supplementary Table 5). The association was rendered non-significant after inclusion of baseline eGFR into the model, highlighting that the ability of the methylation changes to predict incident ESKD was mediated by methylation changes associated with baseline eGFR.

## Discussion

In this study of methylation profiles from a cohort of patients with type 2 diabetes, our major findings are as follows: (1) DNA methylation level was associated with renal function in type 2 diabetes; (2) methylation levels of previously unidentified CpG sites were associated with baseline eGFR; (3) a set of eight previously unidentified CpG sites was associated with the rate of eGFR decline; (4) it is possible to construct prediction models using methylation data for baseline eGFR and decline in eGFR with replication in independent cohorts with type 2 diabetes; (5) proximal genes of the previously unidentified CpG sites and those included in the prediction models were implicated in pathways related to the pathogenesis of kidney diseases; and (6) the prediction models constructed can achieve comparable prediction to models incorporating clinical risk factors. Our study provides insights on the potential of incorporating methylation biomarkers to facilitate risk stratification in type 2 diabetes.

Our results extend earlier work by ourselves and others in highlighting the potential link between kidney function and methylation profile. In particular, the top sites identified in our study, cg17944885, near *ZNF20*, corresponded to a CpG site which had been reported in several EWAS for kidney function^22–25^. Furthermore, CpG sites identified in other studies with methylation levels associated with kidney function in the general population also demonstrated nominal association in our analysis of methylation changes. These results suggested that methylation changes associated with kidney function in the general population may also be applicable to a population with type 2 diabetes. The earlier EWAS were mainly conducted in European populations with subsequent replication in multiethnic cohorts. Together with our results, we might conclude that methylation profiles are not ethnic-specific, as in the case of genetic loci identified from GWAS. Several of our findings were also reported in two recent meta-analysis of EWAS^10,22^, although many of them had not been identified in the earlier individual cohort studies. While this may reflect improved statistical power from the recent larger meta-analysis, a trans-ethnic meta-analysis may be a more powerful strategy for discovering sites that are relevant for different ethnic populations.

In general, there was greater consistency for findings related to methylation changes associated with baseline eGFR compared to decline in kidney function. This is not surprising, given that multiple factors such as blood pressure, lipids, and glycaemia as well as medications might modulate renal and vascular pathology to influence progression of kidney function. Despite the strong association, it is difficult to disentangle the causal relationship between methylation changes and kidney function at baseline. The strong association between baseline eGFR and methylation changes might be consequences of the altered metabolic milieu related to kidney dysfunction. On the other hand, methylation changes predictive of kidney function decline, with minimal overlap with sites associated with baseline eGFR, are more likely to be of use as prognostic biomarkers.

In general, our EWAS results of baseline eGFR were most consistent with those reported by Chu et al. in the ARIC and FHS cohorts^23^ and Breeze et al. in multiple studies and ethnicities^22^. A number of their top sites also had significant *P* values in our data, even though none of these previous studies was conducted on Chinese-specific cohorts or cohorts consisting only of patients with type 2 diabetes (Supplementary Fig. 6, Supplementary Data 1, and Supplementary Data 2). Other than cg17944885, 13 significant CpG sites at FDR = 0.05 in our cohort, including cg25364972, cg02304370, cg12065228, cg21745599, cg16292343, cg05554494, cg22386583, cg09299075, cg13924998, cg07814567, cg03919650, cg19942083, and cg26099045, were also reported as significant signals in either ARIC or FHS cohort, and one significant CpG site in our data, cg23597162, was identified in both the ARIC and FHS cohorts^23^.

Both our single-site and multisite analyses identified cg00506299 as being associated with baseline eGFR. This site is located within the *RFTN1* gene, the methylation level of which has not been previously associated with kidney function. However, *RFTN1* was found differentially expressed between DKD and controls^31^ and correlated with VvInt^29^ in patients with DKD. In FAN mouse kidneys, *Rftn1* was differentially expressed as compared to kidneys from healthy mice^32^. As another example, cg21919729, located within the *CTSB* gene and identified by our single-site analysis, did not have its methylation reported to be associated with kidney disease. However, its expression was correlated with VvInt^29^ in patients with DKD. Its mouse homologous gene *Ctsb* was also differentially expressed in proximal tubule (PT) cells between FAN mice and healthy controls^32^. *CTSB* encodes cathepsin B, a member of the C1 family of peptidases, which produces a lysosomal cysteine protease with both endopeptidase and exopeptidase activity that may play a role in protein turnover. Cathepsin B is involved in inflammation, apoptosis and autophagy during ESKD, chronic kidney disease, and AKI^47^.

Interestingly, the majority of the most significant CpG sites reside in the gene body, highlighting the increasingly recognized role of gene body and non-promoter methylation as important mechanisms of gene regulation in metabolic diseases^48,49^. Among the 74 CpG sites associated with rate of decline in eGFR in people with diabetes in our study, none was reported in previous studies of the general population, which demonstrates the utility of undertaking discovery efforts specifically in people with diabetes to advance precision medicine in diabetes.

From our functional evaluation using other datasets, these CpG sites identified to be associated with diabetic kidney disease point towards other genes implicated in kidney function and kidney diseases, highlighting the potential to use methylation markers in peripheral blood to obtain important biological insights in organ-specific diseases, such as examples from previously noted inter-individual variation in methylation across blood and brain^50^. The fact that methylation level of some of the CpG sites in blood, or in kidneys, show correlation to fibrosis and glomerulosclerosis, further highlight the potential of identifying organ-specific pathology using methylome from peripheral blood.

Although we identified several methylation sites strongly associated with kidney function and decline in kidney function which reached stringent threshold of statistical significance after considering the number of statistical tests, the construction of a prediction model did not necessarily include all of these individually significant CpG sites. This might be because of strong correlation among individual CpG sites, due to spatial dependency or other reasons, leading to redundancy.

In the future, it will be useful to directly test the functional significance of the CpG sites identified in this study in kidney tissues or cell/organoid models. One possible way is to use CRISPR-Cas9-based genome editing to turn a CpG site into a non-CpG sequence, to test the effect of losing CpG methylation at the site. Another possible way is to use CRISPR-based epigenome editing, such as dCas9 fused with a DNA methyltransferase to test the effect of gaining DNA methylation, or dCas9 fused with the ten-eleven translocation (TET) methylcytosine dioxygenase to test the effect of losing DNA methylation.

The prediction model with the best performance included a combination of CpG sites, many of which were not individually strongly associated with eGFR or eGFR decline. The difference in performance between prediction models incorporating multiple sites versus ones including only top individual CpG sites is analogous to the recent development of genome-wide polygenic risk scores^51^. The latter tend to have better performance and utility than the traditional approach of developing polygenic risk scores based on only GWAS-significant hits^52^. Given a large number of methylation datasets currently available, our approach may be applicable for developing other prediction models based on epigenome-wide methylation data, an approach taken by the pioneering work of epigenetic clocks^53^. The prediction model based on our multisite methylation signature also had comparable performance with established risk equations using clinical parameters to predict adverse renal outcome, whereas our data provided additional insights on biological pathways. Our results also suggest that the methylation signature could capture most of the information provided by clinical risk factors, and that inclusion of clinical risk factors did not substantially improve prediction. In contrast, most studies that add genetic variables to clinical markers only marginally improved the prediction of diabetes-related complications^54^. Thus, our results highlight the potential utility of incorporating methylation changes to risk models to improve risk stratification.

Our study has several strengths, including methylation profiling of a moderately large number of subjects with type 2 diabetes with long duration of follow-up for kidney outcomes and assessment of kidney function decline. Most subjects were free of DKD at baseline. We acknowledge several limitations. The discovery was undertaken in a cohort of Chinese patients at comparatively high risk of DKD progression, though the model developed was applied to a group of patients with diabetes in a different clinical setting. In particular, our methylation signature was based on methylation changes in circulating leukocytes as opposed to methylation changes within the kidney. Nevertheless, peripheral blood is a readily accessible tissue for risk stratification in clinical practice, and numerous studies have demonstrated the ability to identify biomarkers in disease-relevant pathways using methylation changes in leukocytes^55^.

Our results highlight the potential utility of using methylation levels in blood samples to predict eGFR or change in eGFR in different populations. We have also identified previously unidentified methylation markers associated with kidney function and decline in kidney function and kidney pathology. Our study highlights the potential of using methylation markers in the risk stratification of renal disease among individuals with type 2 diabetes.

## Methods

### Participant recruitment and clinical variable measurements

We included subjects from HKDR, which was established at the Prince of Wales Hospital, the teaching hospital of the Chinese University of Hong Kong. The HKDR consecutively enrolled patients who were referred to the Diabetes Mellitus and Endocrine Centre for comprehensive assessment of complications and metabolic control, including patients referred from specialty clinics, community clinics and general practitioners^46^. Subjects with diabetes were evaluated as part of a structured assessment for diabetes complications according to a modified European DiabCare protocol. All patients in the HKDR underwent clinical assessments and laboratory investigations after 8-hour overnight fast, including eye, feet, urine, and blood examinations. Eye examination included visual acuity and fundoscopy through dilated pupils or retinal photography. Retinopathy was defined by typical changes due to diabetes, laser scars, or a history of vitrectomy. Foot examination was performed using Doppler ultrasound scan and monofilament and graduated tuning fork. Fasting blood was sampled for measurement of plasma glucose, HbA1c, lipid profile (total cholesterol, high-density lipoprotein [HDL] cholesterol, triglycerides and calculated low-density lipoprotein [LDL] cholesterol), and random spot urinary sample was used to assess albumin to creatinine ratio (ACR). The CKD-EPI equation^56^ was used to estimate glomerular filtration rate.

Ethical approval was obtained from the Joint Chinese University of Hong Kong-New Territories East Cluster Clinical Research Ethics Committee. Written informed consent was obtained from all subjects at the time of enrollment for the collection of clinical information and biosamples for archival and research purposes.

Between 1995 and December 31, 2007, a consecutive cohort consisting of 10,129 patients with diabetes was assessed, with follow-up. Clinical outcomes were defined using hospital discharge diagnoses based on the International Classification of Diseases, Ninth Revision (ICD-9). The Hong Kong Hospital Authority Central Computer System records admissions to all public hospitals, which provides about 95% of inpatient bed-days in Hong Kong. All hospitalization records were retrieved from this system using a unique identifier number. Results of follow-up investigations, including eGFR were likewise retrieved for each subject from the electronic health record from the Central Computer System^57^. For the current analysis, we created a nested case–control cohort based on incident ESKD or incident cardiovascular disease (defined according to the censor date of June 30th, 2017, around the time when the EWAS was initiated and when the case–control status was defined), whereby each subject free of DKD at follow-up was matched with a case of incident ESKD with a similar age at baseline. ESKD was defined by the codes of dialysis (procedure codes 39.95 or 54.98), kidney transplant (procedure code 55.6 or diagnosis codes 996.81 or V42.0), or eGFR<15 ml/min/1.73 m^2^. All subjects were selected based on being free of known cardiovascular events at baseline. In addition to baseline kidney function data, we retrieved follow-up laboratory data through June 30th, 2017, in order to calculate the eGFR slope during follow-up for each individual, up to the censor date, eGFR<15 ml/min/1.73 m^2^, or death, whichever event occurred sooner.

### DNA methylation data production and processing

Whole blood was taken at the baseline assessment visit in a fasting state. Genomic DNA from leukocytes was extracted using traditional phenol-chloroform methods and quantified using Picogreen. Bisulfite conversion was performed using EZGold Methylation kit (Zymo), as per standard protocol. After DNA extraction and bisulfite treatment, DNA methylation in each sample was measured using the Illumina Infinium HumanMethylation450K BeadChip, which covered around 485,000 CpG sites across the genome.

The RnBeads package (version 1.6.1)^58^ was used to preprocess the raw data. First, 10,119 sites were removed due to overlapping with single nucleotide polymorphisms (SNPs). Probes and samples with a large fraction of unreliable measurements, defined as those with detection *P* values larger than 0.05, were also removed. Furthermore, probes in contexts other than CpG sites and probes on sex chromosomes were removed, as was done in some previous studies^11,20^. Background correction was conducted using the “noob” method in the methylumi package (version 2.20.0)^59^ and the signal intensities were normalized using the SWAN method^60^ in the minfi package (version 1.20.2)^61^. After these filtering and normalization steps, 453,128 probes and 1268 samples remained, each quantified by a beta value. In all downstream analyses, we excluded probes with missing methylation values in any sample, resulting in the final number of 434,908 probes. In the whole study, genomic coordinates were based on the reference human genome hg19.

### Modeling the clinical variables using top DNA methylation principal components

Dimensionality reduction of the methylation data was performed using PCA. The top PCs were taken as features of each sample to model each of the clinical variables in a classification setting. Specifically, for each clinical variable, we mapped their values to binary class labels using the criteria listed in Supplementary Table 6. When considering each clinical variable, samples with missing values were omitted. We then constructed logistic regression models with L2 regularization using the Python scikit-learn package (version 0.20.3)^62^ following a 10-fold cross-validation procedure. In this procedure, the whole set of samples was randomly divided into 10 subsets, and each time 9 subsets were used to construct a model while the remaining subset was used to evaluate the model performance, quantified by AUROC. The ten sets of results were then reported separately, together with their mean values. We also tried two other modeling methods, namely support vector classifier with a radial-basis kernel and random forest, and obtained largely comparable results as the logistic regression models (Supplementary Table 7). This same procedure was also used when we modeled eGFR using sex, age, and smoking status alone and with the top PCs.

### Cell-type composition estimation

To adjust for cell heterogeneity of whole-blood samples, cell-type compositions were estimated using a reference-based approach^21^. Using raw methylation data as input, we generated estimated cell counts for CD4 + T cells, CD8 + T cells, NK cells, B cells, monocytes, and granulocytes, using the estimateCellCounts function implemented in the minfi package (version 1.28.4)^61^.

### Single-site epigenome-wide association study (EWAS)

Baseline eGFR was calculated using the Chronic Kidney Disease Epidemiology Collaboration (CKD-EPI) equation^56^. The eGFR slope of each individual was determined by fitting a linear mixed model^63^ and expressed as the percentage change of eGFR per year:

1 $${{\log }}\left({eGF}{R}_{{ij}}\right)={\beta }_{0}+{\beta }_{1}{t}_{{ij}}+{b}_{0i}+{b}_{1i}{t}_{{ij}}+{\epsilon }_{{ij}},$$

where ${{log }}\left({eGF}{R}_{{ij}}\right)$ is the log-transformed eGFR of *i*-th individual at *j*th measurement, ${t}_{{ij}}$ is the time for measuring *eGFR*_*ij*_, ${\beta }_{0}$ and ${\beta }_{1}$ are coefficients for the fixed effects while ${b}_{0i}$ and ${b}_{1i}$ are coefficients for the random effects that are specific to the *i*th individual, and ${\epsilon }_{{ij}}$ is the random noise. After fitting the model, the individual-specific slope is given by the following:

2 $${(eGFR \, slope)}_{i}=({e}^{{\beta }_{1}+{b}_{1i}}-1)\times 100,$$

which is expressed as the percentage change of eGFR per year.

For each CpG site, a linear model was constructed using either baseline eGFR or eGFR slope as the dependent variable and the methylation level as the independent variable. Sex, age, smoking status, duration of diabetes, hemoglobin A1c, blood pressure, experiment batch and the cell-type composition estimations (Supplementary Methods) were included as additional independent variables for models that involved covariates. The *P* value of each CpG site was calculated based on the null hypothesis that it had a zero coefficient in its linear model using two-sided Student’s t test. The Bonferroni procedure was used to perform multiple hypothesis testing correction of the raw *P* values. In addition, the Benjamin–Hochberg procedure was used to identify significant sites at a given false discovery rate (FDR).

### Using *M* values in EWAS

Apart from using beta values to quantify methylation levels, we also used *M* values (where *M* = log_2_ beta/(1-beta)) which yielded similar results. The Pearson correlations of association *P* values of CpG sites with baseline eGFR and eGFR slope were 0.967 and 0.956, respectively. The corresponding Spearman correlations were 0.928 and 0.927 for baseline eGFR and eGFR slope, respectively.

### Multisite models

We also developed a multisite approach that considered all CpG sites at the same time and selected a subset of them to create the best model to infer baseline eGFR or eGFR slope. Briefly, we used LASSO (least absolute shrinkage and selection operator) to construct regression models, which aims at fitting linear models with only a small number of CpG sites having a nonzero coefficient. Performance of each model was evaluated using cross-validation, while the final set of CpG sites (and the corresponding value of the L1 regularization hyperparameter) was selected using a nested procedure that involves the Bayesian Information Criterion (BIC) to balance between model complexity and performance. The constructed models were finally evaluated using left-out testing sets not involved in either training the models or tuning the hyper-parameters.

In detail, we used a multi-step procedure with nested cross-validation to perform model learning, hyperparameter tuning, and unbiased model evaluations (Supplementary Fig. 13). As a data pre-processing step, the methylation levels of each CpG site and the values of each covariate were individually standardized to have zero mean and unit variance.

In our multi-step procedure, we first randomly split the 1268 samples into training (90%) and testing (10%) sets. Using the samples in the training set, we used the tenfold cross-validation procedure to construct linear regression models with LASSO. The value of the regularization parameter $\alpha$ was chosen using grid search based on a nested fivefold cross-validation within each training fold. The value of $\alpha$ chosen (denoted as ${\alpha }^{*}$) for each of the 10 outer training folds was determined using the following criterion:

3 $${\alpha }^{*}={{\max }}\left\{\alpha \, {{\in }}\,{D|}{R}_{\alpha }^{2}{{\ge }}{{\max }}\left({R}^{2}\right)-{SD}\left({R}^{2}\right)\right\},$$

Where ${R}_{\alpha }^{2}$ is the ${R}^{2}$ of the LASSO model using parameter $\alpha$, ${{max }}({R}^{2})$ and ${SD}({R}^{2})$ are the maximum and standard deviation of ${R}^{2}$, respectively, among all the models with different values of $\alpha$ in the set $D$ considered during the grid search. This criterion aims at finding the largest value of $\alpha$ that still gives a model performance close to the one with maximal ${R}^{2}$. The goal of choosing a large value of $\alpha$ is to ensure that only a small set of the most important CpG sites is selected from each model. Using this selected value of $\alpha$, a model was trained with all the samples in the outer training fold. The model was then applied to the samples in the outer testing fold to compute the performance measures. After applying these to all the ten outer training folds, ten sets of performance measures were produced. This whole procedure was further repeated ten times with different random splits of data into ten folds each time, leading to a total of 100 models and correspondingly 100 sets of performance measures.

To produce a single model based on these 100 sets of results, we assigned a weight to each CpG site based on the number of times that it was included in the models and the performance of these models, using the following formula:

4 $${w}_{k}=\mathop{\sum }\limits_{j=1}^{10}\mathop{\sum }\limits_{i=1}^{10}{\rho }_{{ij}}^{{\prime} },$$

5 $${\rho }_{{ij}}^{{\prime} }=\left\{\begin{array}{c}{\rho }_{{ij}},\,{ifCp}{G}_{k}\in {S}_{{ij}}\\ 0,\,{otherwise}\end{array}\right.,$$

where ${w}_{k}$ is the weight of the *k*th CpG site, ${\rho }_{{ij}}$ is the Pearson correlation between prediction and actual values in the *i*th outer testing fold for the *j*th repeat, and ${S}_{{ij}}$ is the set of CpG sites selected by the *i*th outer training fold for the *j*th repeat with a nonzero coefficient. Based on this formula, a CpG site would generally get a higher weight if it has a nonzero coefficient in more models and/or in models that have better performance in terms of Pearson correlation.

All the CpG sites were then sorted in descending order according to their weights. A second series of linear regression models with LASSO were then constructed using different numbers of CpG sites with the largest weights as features with all samples in the original training set for training. The final number of CpG sites to use, ${n}^{*}$, was determined using the following formula that involves the Bayesian Information Criterion (BIC):

6 $${n}^{*}={{\max }}\left\{n|{BI}{C}_{n}\le {{\min }}\left({BIC}\right)+0.1{SD}({BIC})\right\},$$

where ${BI} {{C_n}}$ is the BIC of the model involving the *n*highest-weight CpG sites as features, and ${{\min }}\left({BIC}\right)$ and ${SD}({BIC})$ are the minimum and standard deviation of BIC among all the models with different numbers of CpG sites, respectively. This formula aims at maximizing the number of CpG sites while having a model with a BIC close to the one with the minimal BIC. This time, the number of CpG sites was maximized because the highest-weight CpG sites should already be the most important ones, and including more of them in the model could ensure its robustness. The performance of the model that involved the n highest-weight CpG sites was then evaluated objectively using the original testing set, which was not involved in any training and parameter tuning steps described above.

Finally, all 1268 samples were used together to train a final model for baseline eGFR and another model for eGFR slope, both using the same procedure described above to determine the number of CpG sites. With the selected CpG sites, we trained another version of these two models without including the covariates. Since these final models involved all 1268 samples in model training and parameter tuning, there were no left-out samples in the primary cohort that could independently evaluate their performance.

### Validation of the models in a cohort of Native Americans

Our multisite models were tested in an independent Native American cohort, which contained 326 participants with type 2 diabetes. Baseline eGFR, eGFRs during a mean follow-up of 9.5 years and other clinical variables were measured for each participant. The raw eGFR slope for each subject was calculated using linear regression across all available eGFR measures. The mean baseline eGFR is 106.7 ± 15.1 ml/min/1.73 m^2^. DNA methylation was measured by Illumina Infinium HumanMethylation450K BeadChip and processed as described before^20^. In brief, Minfi^61^ was used to preprocess the data and perform quality control. RnBeads^58^ was then used for beta-mixture quantile normalization.

To use this replication cohort to evaluate the performance of models constructed from the primary cohort, we took the intersection of CpG sites which passed quality control in both cohorts. All samples in the primary cohort were used to learn the baseline eGFR and eGFR slope models based on the subset of CpG sites found in both cohorts, using the same procedure as described above. These models were then applied to the Native American cohort to compare the predicted baseline eGFR or eGFR slope values and their corresponding actual measurements (Fig. 2e–h and Table 4). To check whether our original model is directly applicable to other cohorts, we also applied the original model in the Native American cohort without re-training and performed the same evaluation. All CpG sites not available in the Native American cohort had their methylation levels set to 0 in this case (Supplementary Table 3 and Supplementary Fig. 10). All analyses were performed using R.

### Functional significance of our CpG sites’ methylation levels in kidney samples

Seven CpG sites were selected to check their methylation levels in kidney samples using a published dataset with methylation data from 506 human kidneys^64^. In this dataset, the samples belong to five groups based on the donors’ disease status, namely Con (normal kidneys, 113 samples), CKD (eGFR <60, 101 samples), DKD (having both CKD and diabetes, 63 samples), DM (having diabetes but not CKD, 97 samples), and HTN (having hypertension but not CKD, 132 samples).

Among the seven CpG sites selected for lookup, one (cg21573651) was associated with both baseline eGFR and eGFR slope in the single-site analysis. The other six CpG sites (cg17944885, cg06449934, cg02304370, cg21919729, cg04610187 and cg18593194) were associated with baseline eGFR and were the top six sites among the 36 CpG sites identified in both single-site and multisite analyses.

### Morphometric analysis in kidney biopsies of patients with type 2 diabetes

To evaluate links between blood methylation and structural changes in the kidney, we utilized data from analyses of the morphometric variables from the kidney biopsies from 84 individuals who had biopsies done and methylation in peripheral blood has been measured using the Illumina Infinium HumanMethylation450 array in the same individuals^20,65^. Results are reported as partial correlation and *P* value for each of the nine key variables. The parameters evaluated included: FPW—podocyte foot process width (nm) (higher is worse), GBM—glomerular basement membrane width (nm) (higher is worse), GS—global glomerular sclerosis (%) (higher is worse), V_G_—mean glomerular volume (×10^6^ µm^3^) (higher is worse), non-Podo—mean non-podocyte number per glomerulus (N) (lower is worse), Fen—percent fenestrated endothelium (%) (higher is worse), S_V_ (glomerular filtration surface density) (µ^2^/µ^3^) (lower is worse), VvInt—cortical interstitial fractional volume (%) (higher is worse), VvMes—mesangial fractional volume (%) (higher is worse). These morphometric parameters were selected because they were previously associated with loss of kidney function in this cohort^29,66^.

### Risk equations comparison

To calculate the eGFR of each subject five years after the baseline measurements using the eGFR slope determined by Eqs. (1) and (2), the following formula is used:

7 $${c}_{i}={\beta }_{1}+{b}_{1i}={{\log }}\left(\frac{{\left({eGFR}\,{slope}\right)}_{i}}{100}+1\right),$$

8 $${\left({eGFR}\right)}_{i5}={\left({eGFR}\right)}_{i0}\times {e}^{5{c}_{i}},$$

where ${\left({eGFR}\right)}_{i0}$ and ${\left({eGFR}\right)}_{i5}$ are the eGFR of *i*-th individual at baseline and five years after the baseline, respectively. We defined subject *i* to have ESKD in five years after the baseline if ${\left({eGFR}\right)}_{i5}$ < 15 ml/min/1.73 m^2^.

For each patient, the actual ESKD status was determined using the above method based on his/her actual eGFR slope obtained by making use of all his/her eGFR measurements during the follow-up period. Similarly, the ESKD status predicted by our model was produced using the above method based on the predicted eGFR slope, the multisite model of which was constructed using DNA methylation. This was achieved by a fivefold cross-validation procedure, in which every time 4/5 of the patients were used to train the multisite model, which was applied to the remaining 1/5 of the patients to predict their 5-year ESKD status. The risk scores of the risk equations for renal outcomes by JADE risk model^41,46^ and UKPDS-OM2^44^ were calculated following the descriptions in the original publications.

An independent nested case–control cohort of 181 individuals with type 2 diabetes, of which 80 developed ESKD during follow-up^20^, were included to examine association between blood methylation level and progression to ESKD.

### Inclusion and ethics statement

In this research study, local researchers were included throughout the research process, including study design, study implementation, data ownership, intellectual property, and authorship of the publication. This study is locally relevant and has been determined in collaboration with local partners. Roles and responsibilities were agreed among collaborators ahead of the research. This study was approved by a local ethics review committee. Local and regional research relevant to this study has been taken into account in citations.

The original discovery analysis in Hong Kong Diabetes Register is supported by ethics approval from the Joint Chinese University of Hong Kong-New Territories East Cluster Clinical Research Ethics Committee. Written informed consent was obtained from all participants.

The validation of the models in a cohort of Native Americans was conducted by the NIDDK Intramural Research Program, and these protocols were approved by the NIDDK/NIH Institutional Review Board. Written informed consent was obtained from all participants.

### Reporting summary

Further information on research design is available in the Nature Portfolio Reporting Summary linked to this article.

## Supplementary information

Supplementary Information

Description of Additional Supplementary Files

Supplementary Data 1

Supplementary Data 2

Supplementary Data 3

Supplementary Data 4

Reporting Summary

## Data Availability

Individual-level data are protected and are not available because of ethical restriction, as they were not consented for sharing on a public platform. Summary methylation data are available for analysis by qualified researchers who fulfill the criteria for access by providing a copy of the research proposal and analysis plan, proof of ethics approval for the planned methylation analysis, and institutional endorsement of server data security. Readers and colleagues who are interested to obtain further information about the study can contact the Hong Kong Institute of Diabetes and Obesity, The Chinese University of Hong Kong, Hong Kong at hkido@cuhk.edu.hk. The summary statistics of significant CpG sites and the multisite models generated in this study are provided in the Supplementary Information. We have also created a web-based tool using Shiny app so that readers can use the tool to calculate eGFR and eGFR slope based on methylation data, or perform lookup of association between CpG methylation and eGFR. The tool can be accessed at http://hkdbrmlab.shinyapps.io/DKD_EWAS/. Contact person for the Hong Kong Diabetes Register: Professor Ronald Ma, rcwma@cuhk.edu.hk. Contact person for the Native American cohorts: Dr Robert Hanson, rhanson@phx.niddk.nih.gov. Source data are provided with this paper.

## Source data

Source Data

## Code availability

Source code for single-site and multisite analyses can be accessed from our GitHub repository, https://github.com/kellyliyichen/eGFR_450k and Zenodo^67^, under GPL-3.0 license.
