# Supplementary material for: DNA methylation markers for kidney function and progression of diabetic kidney disease
Source: Nat Commun. 2023 May 15;14:2543. doi: 10.1038/s41467-023-37837-7 (PMC10185566; doi:10.1038/s41467-023-37837-7)
Supplement: Supplementary file 1 — Supplementary Information [file 41467_2023_37837_MOESM1_ESM.pdf]

DNA methylation markers for kidney function and progression of diabetic kidney disease

Kelly Yichen Li, Claudia Ha Ting Tam, Hongbo Liu, Samantha Day, Cadmon King Poo Lim, Wing Yee So, Chuiguo Huang, Guozhi Jiang, Mai Shi, Heung Man Lee, TRANSCEND Consortium, Hui-yao Lan, Cheuk-Chun Szeto, Robert L. Hanson, Robert G. Nelson, Katalin Susztak, Juliana C. N. Chan, Kevin Y. Yip*, and Ronald C.W. Ma*

Supplementary Materials

Table of Contents

Full list of consortium members **........ 3**

Supplementary results **........ 4**

Evaluation of data reproducibility 4

Principal component analysis 4

Performance of the multi-site models at different feature selection thresholds 4

Comparing our multi-site models with alternative models 5

Supplementary tables **........ 6**

Supplementary Table 1: Clinical characteristics of the participants in the primary cohort 6

Supplementary Table 2: Performance of classification models using different sets of CpG sites as input 7

Supplementary Table 3: Performance of the multi-site models constructed from data of the primary cohort, using CpG sites available to the primary cohort, and applied to the Native American cohort 8

Supplementary Table 4: Correlation between DNA methylation levels of our seven selected CpG sites in blood and morphometric variables from kidney biopsies in the same individuals 9

Supplementary Table 5: Associations of baseline methylation score with incident ESRD in Native American nested case-control study 10

Supplementary Table 6: Criteria for defining binary classes for clinical variables 11

Supplementary Table 7: Mean AUROCs of different models using top 50 PCs for classifying clinical variables 12

Supplementary Table 8: Performance of regression models using different sets of CpG sites as input 13

Supplementary figures *14*
Supplementary Figure 1: Distributions of eGFR of the subjects 14
Supplementary Figure 2: Evaluation of data reproducibility 15
Supplementary Figure 3: Receiver-operator characteristics of the regularized logistic regression models for sex (a), age (b) and smoking status (c) constructed from the top 50 PCs of DNA methylation 16
Supplementary Figure 4: Receiver-operator characteristics of the regularized logistic regression models for eGFR constructed from the top 50 PCs of DNA methylation alone (a), sex, age, and smoking status alone (b), or both (c) 17
Supplementary Figure 5: Receiver-operator characteristics of the regularized logistic regression models for the other clinical variables constructed from the top 50 PCs of DNA methylation, including duration of diabetes (a), BMI (b), HbA1c (c), FBG (d), LDL (e), HDL (f), TG (g), ACR (h), BP (i), HB (j), LLD (k), ACEI (l), use of insulin (m), and use of anti-hypertensive drugs (n) 18
Supplementary Figure 6: Statistical significance, in our data set, of CpG sites reported in previous studies, including Breeze et al. (a-d), Chen et al. (e), Chu et al. (f-g), Gluck et al. (h), Qiu et al. (i-j), Sheng et al. (k-l), and Schlosser et al. (m) 19
Supplementary Figure 7: Correlation of methylation levels among the significantly associated CpG sites at FDR=0.05 selected by the single-site analysis 20
Supplementary Figure 8: Performance of the multi-site models with different number of CpG sites 21
Supplementary Figure 9: Performance of the multi-site models with the same number of CpG sites as in the real models but randomly selected 22
Supplementary Figure 10: Performance of the multi-site models constructed from data of the primary cohort, using CpG sites available to the primary cohort, and applied to the Native American cohort, for baseline eGFR and eGFR slope with covariates in the inputs (a-b) or without (c-d) 23
Supplementary Figure 11: Random CpG sites near genes related to kidney function 24
Supplementary Figure 12: Functional Significance of our selected CpG sites' methylation levels in kidney 25
Supplementary Figure 13: Training, parameter tuning and evaluation procedures of the multi-site model 26
Supplementary Figure 14: Cumulative variance explained by the top PCs of the methylation data 27
Supplementary Figure 15: AUROC values of the regularized logistic regression models for the four clinical variables most associated with DNA methylation at different number of PCs, including sex (a), age (b), smoking (c), and eGFR (d) 28
Supplementary Figure 16: Comparing the multi-site models with alternative models 29

Full list of consortium members

TRANSCEND (TRansomics ANalysis of Complications and ENdpoints in Diabetes) Consortium Group Members

Ronald C.W. Ma[1,2,3,4], Juliana C.N. Chan[1,2,3], Yu Huang[5], Hui-yao Lan[1,3], Si Lok[3], Brian Tomlinson[1], Stephen K.W. Tsui[5], Weichuan Yu[6], Kevin Y. Yip[7], Ting-fung Chan[8], Xiaodan Fan[9], Wing-yee So[1,2], Cheuk-chun Szeto[1], Nelson L.S. Tang[3], Andrea O.Y. Luk[1,2,3], Xiaoyu Tian[5], Claudia H.T. Tam[1,2], Guozhi Jiang[1,2], Heung Man Lee[1], Cadmon K.P. Lim[1,2], Katie K.H. Chan[2], Fangying Xie[1], Alex C.W. Ng[1], Grace P.Y. Cheung[1], Ming-wai Yeung[1], Shi Mai[5], Fei Xie[1], Wei Jiang[6], Sen Zhang[6], Pu Yu[6], Meng Weng[6], Kelly Yichen Li[7], Chuiguo Huang[1], Gechang Yu[1]

[1] Department of Medicine and Therapeutics, The Chinese University of Hong Kong, Hong Kong

[2] Hong Kong Institute of Diabetes and Obesity, The Chinese University of Hong Kong, Hong Kong

[3] Li Ka Shing Institute of Health Sciences, The Chinese University of Hong Kong, Hong Kong

[4] Integrated Bioinformatics Laboratory for Cancer Biology and Metabolic Diseases, The Chinese University of Hong Kong, Hong Kong

[5] School of Biomedical Sciences, The Chinese University of Hong Kong

[6] Department of Electronic and Computer Engineering, The Hong Kong University of Science and Technology, Hong Kong

[7] Department of Computer Science and Engineering, The Chinese University of Hong Kong, Hong Kong

[8] School of Life Sciences, The Chinese University of Hong Kong, Hong Kong

[9] Department of Statistics, The Chinese University of Hong Kong, Hong Kong

Supplementary results

Evaluation of data reproducibility

For 12 patients, methylation levels were measured independently from 2 technical replicates. Beta values among replicate samples had a median Pearson correlation of 0.998 and these correlation values were significantly higher than those among random sample pairs (Supplementary Figure 2; $p=2.51\times10^{-9}$, two-sided Wilcoxon rank-sum test), indicating high reproducibility of the data.

Principal component analysis

To investigate whether global DNA methylation trends are associated with clinical variables, we performed PCA of the methylation data. The top 50 PCs explained 45% of the total data variance (Supplementary Figure 14), which provide a reasonable tradeoff between retaining a small number of top PCs and a fair amount of variance captured by them. We then constructed a regularized logistic regression model for each clinical variable using top 50 PCs as independent variables. Using the 10-fold cross-validation procedure (Supplementary Methods), we trained the model and evaluated its performance on mutually exclusive subsets of samples. The models with highest cross-validation performance were those for sex (mean area under the receiver-operator characteristics curve [AUROC] of the 10 testing sets=0.99), age (mean AUROC=0.95) and smoking status (mean AUROC=0.82). These results were robust across different sets of training samples (Supplementary Figure 3) and are consistent with previous reports that DNA methylation is highly associated with sex[56,59,60], age[45,61,62] and smoking[63,64].

To test whether the association between DNA methylation and baseline eGFR was due to confounding factors caused by sex, age, or smoking status, we constructed models of baseline eGFR using these three variables alone. We found that the AUROC values were close to the expected value of 0.5 for a random model (Supplementary Figure 4b), showing that baseline eGFR was not influenced by these variables. The resulting AUROC of a model including the 50 top PCs of DNA methylation and these three top variables (sex, age, smoking status) was not higher than the one with only the 50 PCs (Supplementary Figure 4c). These results indicated that the fairly strong association between baseline eGFR and global DNA methylation trends was independent of other clinical variables.

We repeated the modeling procedures using other numbers of top methylation PCs as features (Supplementary Figure 15). For the models for baseline eGFR, similar to those for age and smoking status, the mean AUROC value generally displayed a decreasing trend as more PCs were included, showing that the most accurate models could be obtained by considering only a small number of the most informative features.

Performance of the multi-site models at different feature selection thresholds

Supplementary Figure 8 shows the performance of the models at different feature selection thresholds as evaluated by the overall testing set. In general, when a less stringent feature selection threshold was used, more CpG sites would

be included in the models and the training performance would be higher, yet the performance on the left-out testing sets was not necessarily better. This inconsistency indicates that overfitting could have occurred when the models contained too many CpG sites. This observation also confirms the importance of evaluating the models using data not involved in model training. For both baseline eGFR and eGFR slope, the maximal modeling performance, as judged by both the Pearson correlation between the actual and inferred values or their mean squared error computed from the left-out testing data, could be achieved with a stringent feature selection threshold by including a small number of CpG sites. This is similar to the situation with the PCA results where including too many components tended to reduce the performance.

Comparing our multi-site models with alternative models

To further evaluate the importance of these CpG sites that were individually not strongly associated with the target traits, we compared our final models with three alternative models constructed with different choices of input CpG sites. They included 1) a subset of sites in our final models with a single-site Bonferroni-corrected p-value < 0.05, 2) a subset of sites in our final models with significance at FDR=0.05 in the single-site analysis, and 3) sites with the most significant single-site p-values among all CpG sites, with the total number of sites being the same as our final models (64 for baseline eGFR and 37 for eGFR slope). All these alternative models did not perform as well as our original models (Supplementary Figure 16, Supplementary Table 8), showing that the auxiliary CpG sites played crucial roles in modeling baseline kidney function and its decline over time.

Supplementary tables

Number of samples before filtering	1,271
Number of samples after filtering	1,268
Baseline characteristics	
Male % (N)	50.6% (642)
Age (years)	57.1 ± 11.3
Age of diabetes onset (years)	49.2 ± 11.5
Duration of diabetes (years)	7.9 ± 6.9
Smoking status % (N)	
Non-smoker	69.4% (878)
Ex-smoker	16.7% (212)
Current smoker	13.9% (176)
Body height (m)	1.59 ± 0.08
Body weight (kg)	63.5 ± 11.9
Body mass index (kg/m^2)	25.1 ± 3.9
Waist circumference (cm)	
Male	87.7 ± 9.1
Female	84.0 ± 9.8
Hip circumference (cm)	96.3 ± 7.9
Waist-hip-ratio	0.9 ± 0.1
HbA1c (%)	7.9 ± 1.9
Total cholesterol (mmol/L)	5.4 ± 1.3
Triglycerides (mmol/L)	1.4 (1.0 – 2.2)
HDL-cholesterol (mmol/L)	1.3 ± 0.4
LDL-cholesterol (mmol/L)	3.3 ± 1.11
Systolic blood pressure (mm Hg)	137 ± 20.5
Diastolic blood pressure (mm Hg)	77.3 ± 11.1
Hypertension % (N)	74.2% (941)
Retinopathy % (N)	31.2% (396)
Neuropathy % (N)	23.1% (293)
Microalbuminuria % (N)	23.1% (283)
Macroalbuminuria % (N)	21.8% (268)
Albumin-creatinine-ratio	2.3 (0.8 – 17.4)
eGFR (ml/min/1.73 m^2) – CKD-EPI	80.6 ± 25.0
Treatment	
Lipid lowering drug % (N)	13.8% (175)
Blood pressure anti-hypertensive drug % (N)	41.7% (529)
ACE inhibitor/ARB % (N)	20.0% (253)
Oral glucose lowering drug % (N)	61.5% (780)

Data are shown as either a single value and the corresponding percentage of individuals with measurements, mean value ± standard deviation, or median and the corresponding inter-quartile range between the first and third quartiles. Some variables (e.g., smoking status) contained some missing values.

Supplementary Table 1: Clinical characteristics of the participants in the primary cohort

Input CpG sites	Covariates	mean AUROC
Baseline eGFR		
All	Yes	0.893
	No	0.883
Corrected p<0.05	Yes	0.885
	No	0.825
Significant at FDR=0.05	Yes	0.897
	No	0.876
Most significant	Yes	0.875
	No	0.841
Covariates only	Yes	0.832
eGFR slope		
All	Yes	0.805
	No	0.780
Corrected p<0.05	Yes	0.756
	No	0.627
Significant at FDR=0.05	Yes	0.782
	No	0.706
Most significant	Yes	0.772
	No	0.701
Covariates only	Yes	0.750

The input CpG sites of the alternative models are defined in the Supplementary Results section. Binary class threshold is 60 and -4 for baseline eGFR and eGFR slope, respectively. All results shown here were determined based on 10-fold cross-validation (stratified with class labels).

Supplementary Table 2: Performance of classification models using different sets of CpG sites as input

Testing cohort	Target phenotype	No. of CpG sites in the original model	No. of used CpG sites	% of CpG used sites	Covariates	PCC	SCC	MAE
Native Americans	Baseline eGFR	55	51	92.7	Yes	0.856	0.621	26.997
		55	50	90.9	No	0.491	0.540	27.614
	eGFR slope	32	31	96.9	Yes	0.324	0.360	4.284
		36	35	97.2	No	0.237	0.261	4.295

The "No. of CpG sites in the original model" column shows the number of CpG sites with non-zero coefficients in the original model constructed using the primary cohort. The "No. of used CpG sites" column shows the number of CpG sites in the model that are present in the Native American cohort. PCC: Pearson correlation coefficient, SCC: Spearman correlation coefficient, MAE: mean absolute error.

Supplementary Table 3: Performance of the multi-site models constructed from data of the primary cohort, using CpG sites available to the primary cohort, and applied to the Native American cohort

	cg21573651	cg17944885	cg06449934	cg02304370	cg21919729	cg04610187	cg18593194
r_FPW	0.04	-0.19	-0.05	0.01	-0.08	0.12	-0.23
p_FPW	0.74	0.12	0.70	0.95	0.50	0.34	0.07
r_GBM	-0.08	0.01	-0.09	-0.06	0.05	0.10	0.04
p_GBM	0.52	0.96	0.45	0.62	0.68	0.44	0.74
r_GS	0.04	-0.14	-0.06	-0.29	0.04	-0.07	-0.25
p_GS	0.76	0.25	0.63	**0.01**	0.75	0.55	**0.03**
r_V_G	0.06	-0.05	0.14	-0.03	0.12	0.08	0.10
p_V_G	0.64	0.68	0.23	0.77	0.30	0.49	0.38
r_non-Podo	0.01	-0.04	0.13	-0.03	0.06	0.09	0.10
p_non-Podo	0.92	0.75	0.27	0.82	0.62	0.47	0.39
r_Fen	0.08	-0.01	-0.17	0.01	0.14	-0.06	0.14
p_Fen	0.51	0.95	0.15	0.92	0.24	0.60	0.25
r_S_V	-0.08	0.20	0.04	0.05	0.05	0.05	0.08
p_S_V	0.49	0.10	0.76	0.69	0.67	0.68	0.50
r_VvInt	0.08	0.03	-0.02	-0.05	-0.08	0.00	0.00
p_VvInt	0.52	0.78	0.88	0.66	0.51	0.98	1.00
r_VvMes	-0.10	0.00	0.04	0.08	0.12	0.07	0.00
p_VvMes	0.38	0.97	0.72	0.50	0.34	0.59	0.99

For each variable, the first row (with prefix "r_" added to the variable name) shows the partial Pearson correlations and the second row (with prefix "p_" added to the variable name) shows the p-values. P-values smaller than or equal to 0.05 are in bold face. FPW: podocyte foot process width (nm), GBM: glomerular basement membrane width (nm), GS: global glomerular sclerosis (%), V_G: mean glomerular volume ($\times 10^6$ μm^3), non-Podo: mean non-podocyte number per glomerulus (N), Fen: percent fenestrated endothelium (%), S_V: glomerular filtration surface density (μ^2/μ^3), VvInt: cortical interstitial fractional volume (%), VvMes: mesangial fractional volume (%).

Supplementary Table 4: Correlation between DNA methylation levels of our seven selected CpG sites in blood and morphometric variables from kidney biopsies in the same individuals

Target phenotype	Base model HR (95% CI)	p-value	Base model + baseline eGFR HR (95% CI)	p-value
Baseline eGFR, without covariates	0.59 (0.41, 0.84)	0.0037	1.01 (0.66, 1.54)	0.9714
Baseline eGFR, with covariates	0.66 (0.49, 0.90)	0.0078	1.04 (0.73, 1.49)	0.8188
eGFR slope, without covariates	0.75 (0.58, 0.97)	0.0307	0.90 (0.67, 1.20)	0.4767
eGFR slope, with covariates	0.77 (0.60, 1.00)	0.0518	0.94 (0.71, 1.26)	0.6807

Based on nested case-control study with 80 incident ESRD cases and 181 total individuals[14]. Methylation score for baseline eGFR is based on 64 available CpG sites, while the score for eGFR slope is based on 37 available CpG sites. Hazard ratios (HR) are expressed per SD of the methylation. P-values were computed using Cox proportional hazard model based on the null hypothesis that HR=1. Correlations with baseline eGFR are 0.69 and 0.64 for baseline eGFR target methylation score with and without covariates respectively; corresponding correlations for the eGFR slope methylation score are 0.22 and 0.26, respectively.

Supplementary Table 5: Associations of baseline methylation score with incident ESRD in Native American nested case-control study

Clinical variable	Class 0	Class 1
Sex	Male	Female
Age (years)	<40	≥40
Duration of diabetes (years)	<10	≥10
BMI (kg/m^2)	<25	≥25
HbA1c (%)	<7	≥7
FBG (mmol/L)	<7	≥7
Smoking	CS	NS or ES
LDL (mmol/L)	<2.6	≥2.6
HDL (mmol/L)		
Female:	<1.3	≥1.3
Male:	<1.0	≥1.0
TG (mmol/L)	<1.7	≥1.7
eGFR (ml/min/1.73 m^2)	<60	≥60
ACR	<30	≥30
BP (mm Hg)	SBP<130 and DBP<80	SBP≥130 or DBP≥80
HB (g/dL)		
Female:	<11	≥11
Male:	<13	≥13
Use of LLD	Yes	No
Use of RASi	Yes	No
Use of insulin	Yes	No
Use of anti-hypertensive drugs	Yes	No

BMI: body mass index; FBG: fasting blood glucose; CS: current smokers; NS: non-smokers; ES: ex-smoker; LDL: LDL-cholesterol; HDL: HDL-cholesterol; TG: triglycerides; ACR: albumin-creatinine-ratio; BP: blood pressure; SBP: systolic blood pressure; DBP: diastolic blood pressure; HB: hemoglobin; LLD: lower-lipid drugs. RASi: ACEI/ARB drugs.

Supplementary Table 6: Criteria for defining binary classes for clinical variables

Clinical variables	Mean AUROC		
	LR	SVM	RF
Sex	0.99	0.98	0.99
Age	0.95	0.82	0.86
Duration of diabetes	0.52	0.54	0.52
BMI	0.48	0.48	0.49
HbA1c	0.57	0.55	0.57
FBG	0.45	0.51	0.50
Smoking	0.82	0.69	0.73
LDL	0.57	0.53	0.52
HDL	0.60	0.57	0.59
TG	0.54	0.52	0.50
eGFR	0.76	0.71	0.71
ACR	0.64	0.54	0.61
BP	0.59	0.55	0.56
HB	0.66	0.52	0.63
Use of LLD	0.54	0.49	0.49
Use of RASi	0.46	0.44	0.43
Use of insulin	0.56	0.52	0.52
Use of anti-hypertensive drugs	0.55	0.55	0.52

LR: logistic regression; SVM: support vector machine; RF: random forest.

Supplementary Table 7: Mean AUROCs of different models using top 50 PCs for classifying clinical variables

Input CpG sites	Covariates	PCC	SCC	MAE
Baseline eGFR				
All	Yes	0.762	0.718	12.598
	No	0.719	0.672	13.644
Corrected p<0.05	Yes	0.699	0.674	13.986
	No	0.551	0.492	16.990
Significant at FDR=0.05	Yes	0.743	0.702	13.078
	No	0.662	0.593	14.955
Most significant	Yes	0.715	0.681	13.751
	No	0.600	0.533	16.141
Covariates only	Yes	0.621	0.624	14.973
eGFR slope				
All	Yes	0.551	0.502	4.427
	No	0.528	0.470	4.541
Corrected p<0.05	Yes	0.399	0.380	4.822
	No	0.219	0.200	5.425
Significant at FDR=0.05	Yes	0.451	0.444	4.648
	No	0.343	0.321	5.080
Most significant	Yes	0.450	0.453	4.619
	No	0.339	0.343	5.054
Covariates only	Yes	0.368	0.369	4.871

The input CpG sites of the alternative models are defined in the Supplementary Results section. All results shown here were determined based on 5-fold cross-validation. PCC: Pearson correlation coefficient; SCC: Spearman correlation coefficient; MAE: mean absolute error.

Supplementary Table 8: Performance of regression models using different sets of CpG sites as input

Supplementary figures

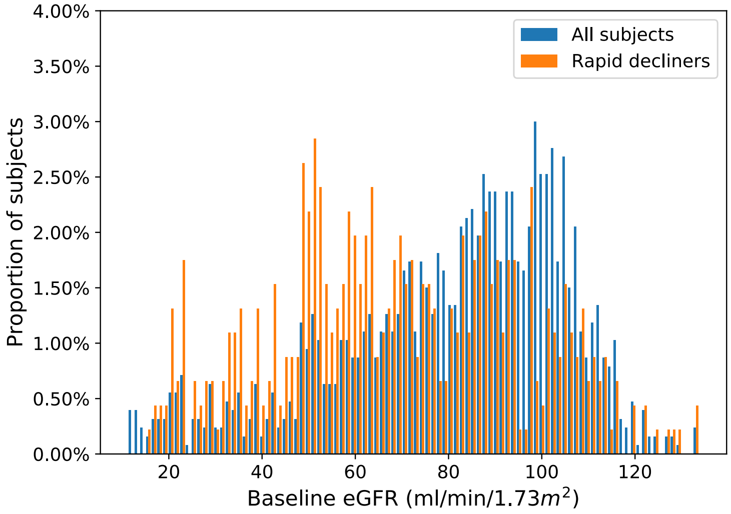

Supplementary Figure 1: Distributions of eGFR of the subjects

Histogram of baseline eGFR in all subjects (blue) and rapid decliners (defined as subjects with eGFR slope ≤ -4% change of eGFR per year) (orange).

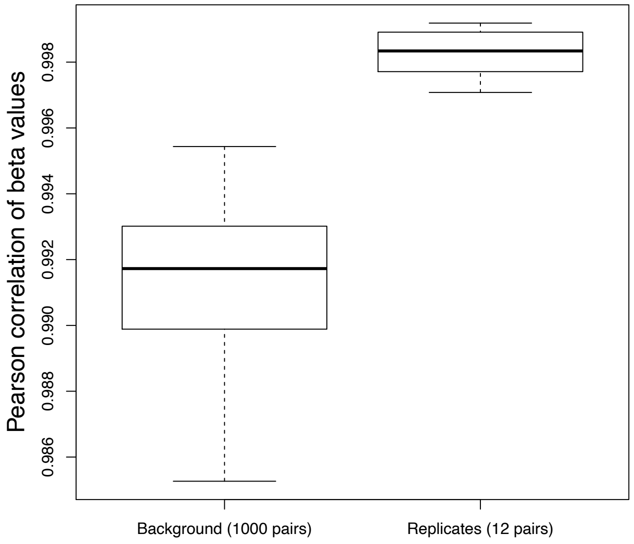

Supplementary Figure 2: Evaluation of data reproducibility

For 12 patients, methylation levels were measured independently from 2 technical replicates. For each pair of replicated samples, the correlation of their beta values across all CpG sites was computed. The distribution of these 12 correlation values is compared with one formed by a background with 1,000 random pairs of samples. In each boxplot, the center lines show the median, and the box limits show the first (Q1) and third (Q3) quartiles (25% and 75%, respectively). Whiskers represents data within the range of Q1-1.5×interquartile range (IQR) ~ Q3+1.5×IQR. Source data are provided as a Source Data file.

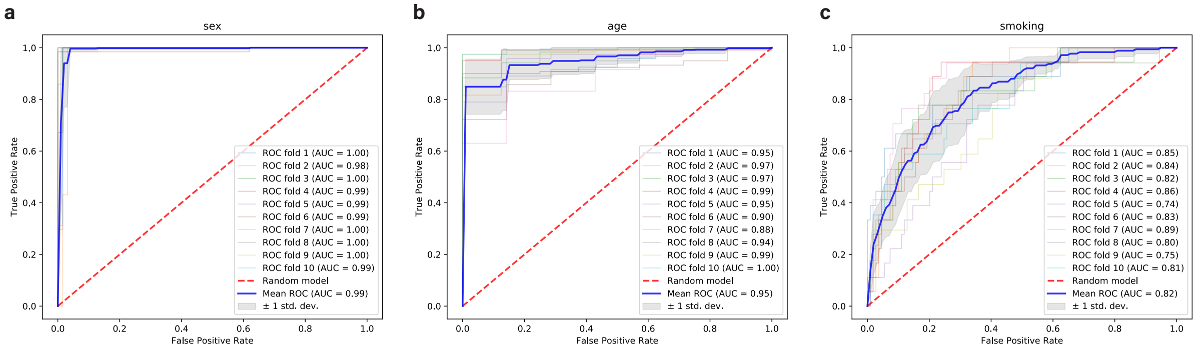

Supplementary Figure 3: Receiver-operator characteristics of the regularized logistic regression models for sex (a), age (b) and smoking status (c) constructed from the top 50 PCs of DNA methylation

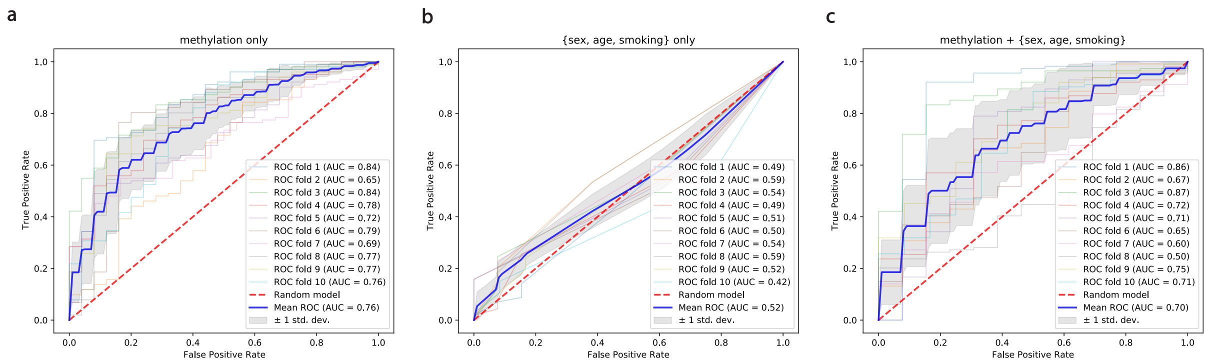

Supplementary Figure 4: Receiver-operator characteristics of the regularized logistic regression models for eGFR constructed from the top 50 PCs of DNA methylation alone (a), sex, age, and smoking status alone (b), or both (c)

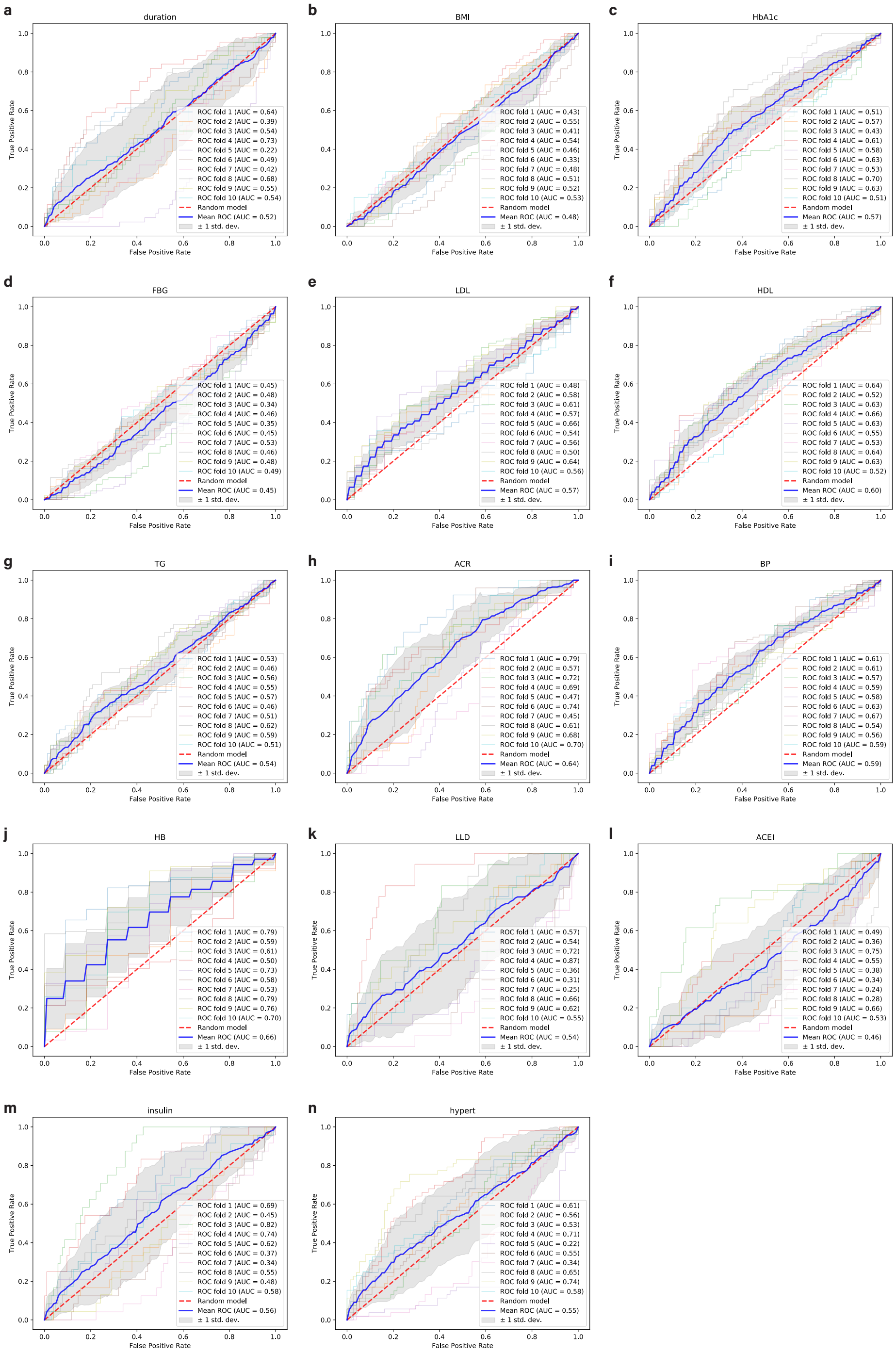

Supplementary Figure 5: Receiver-operator characteristics of the regularized logistic regression models for the other clinical variables constructed from the top 50 PCs of DNA methylation, including duration of diabetes (a), BMI (b), HbA1c (c), FBG (d), LDL (e), HDL (f), TG (g), ACR (h), BP (i), HB (j), LLD (k), ACEI (l), use of insulin (m), and use of anti-hypertensive drugs (n)

LLD: use of lower-lipid drugs; ACEI: use of ACEI/ARB drugs. Other abbreviations are defined in the caption of Supplementary Table 6.

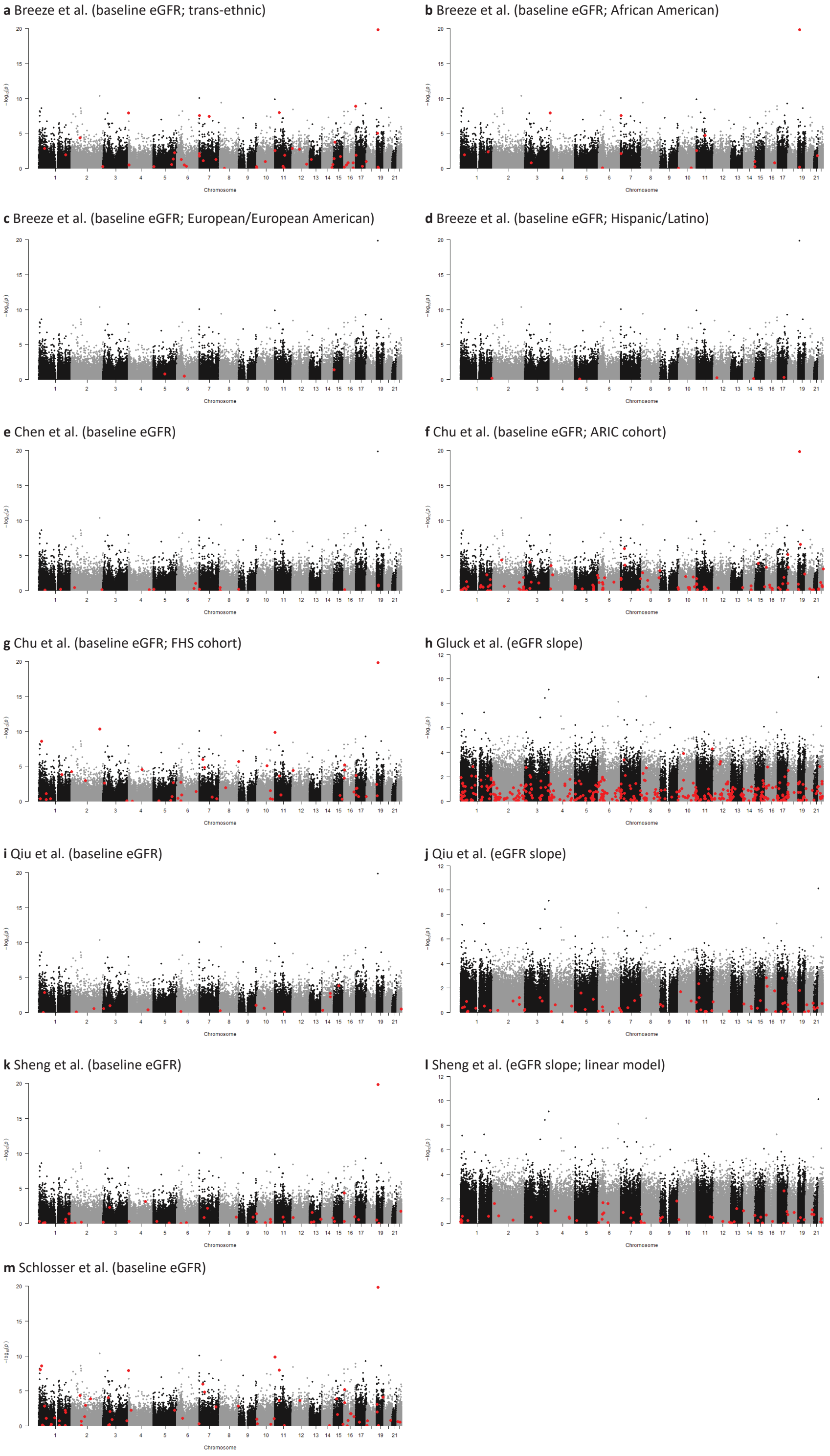

Supplementary Figure 6: Statistical significance, in our data set, of CpG sites reported in previous studies, including Breeze et al. (a-d), Chen et al. (e), Chu et al. (f-g), Gluck et al. (h), Qiu et al. (i-j), Sheng et al. (k-l), and Schlosser et al. (m)

All panels show the same genomic locations and association p-values of the CpG sites in our study, with each panel highlighting the CpG sites reported in a particular previous study in red. P-values were computed using two-sided Student's t test.

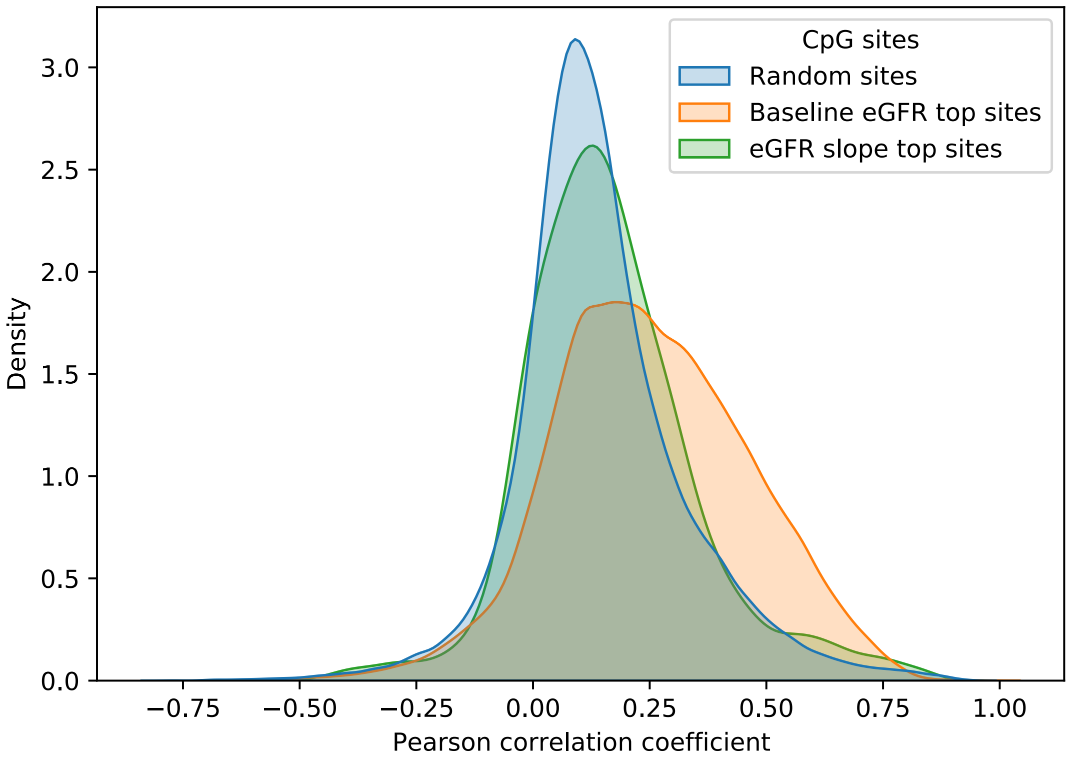

Supplementary Figure 7: Correlation of methylation levels among the significantly associated CpG sites at FDR=0.05 selected by the single-site analysis

The orange and green curves show the distributions of pairwise Pearson correlation coefficients of methylation levels among the top sites for baseline eGFR and eGFR slope, respectively. The blue curve shows the background distribution, formed by randomly sampling 100,000 pairs of CpG sites.

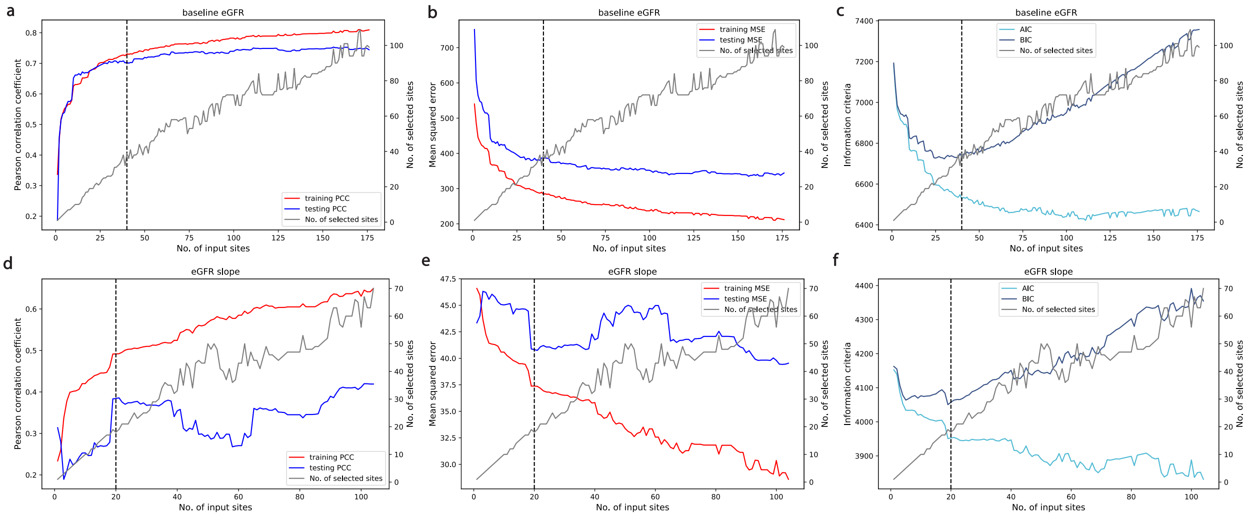

Supplementary Figure 8: Performance of the multi-site models with different number of CpG sites

The performance of the models for baseline eGFR (a-c) and eGFR slope (d-f) was evaluated based on the Pearson correlation between the model outputs and the actual values (a,d) and the mean squared error between them (b,e), and the number of CpG sites selected as input to enter the final model was determined based on information content (c,f). In each panel, the x-axis shows the number of top CpG sites selected by the procedure for constructing the model, while the gray curve shows that actual number of CpG sites with a non-zero coefficient. The vertical dotted lines show the final models determined according to the information content.

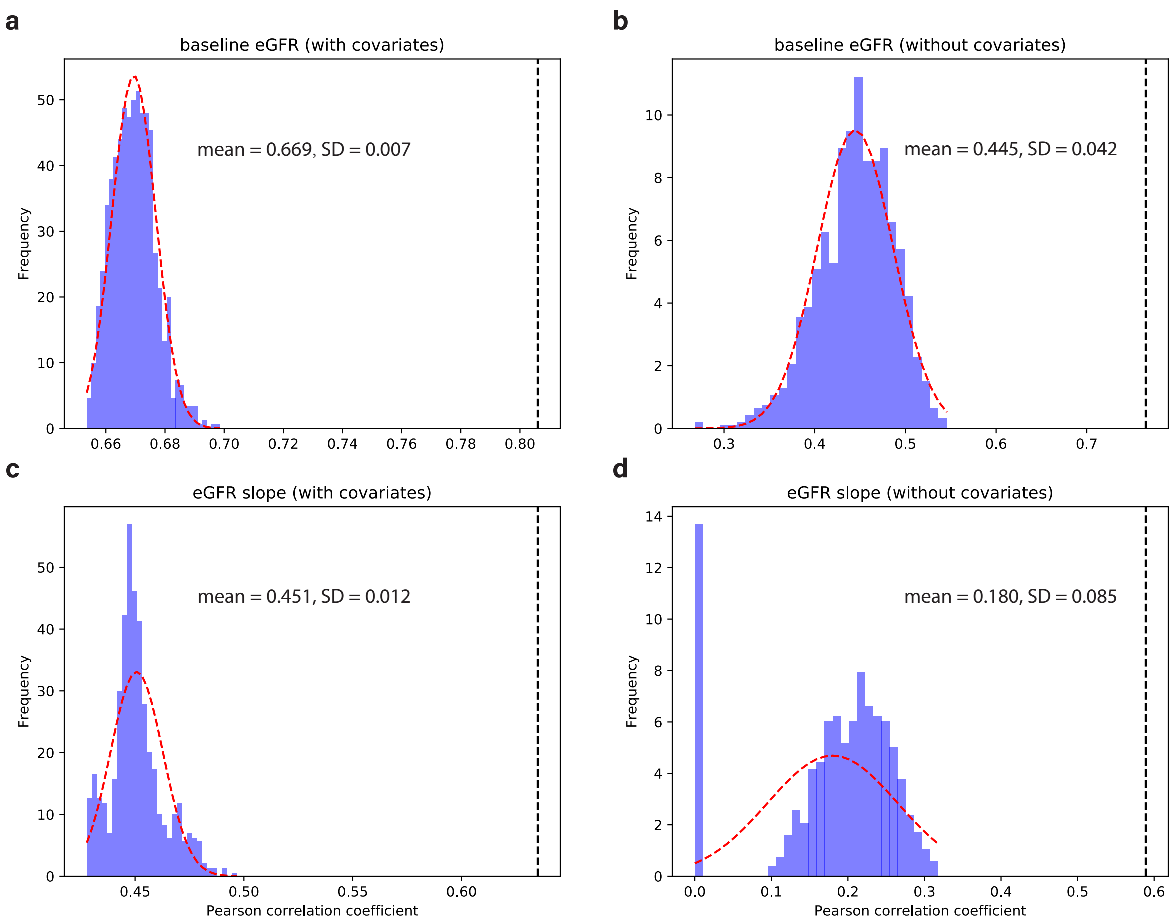

Supplementary Figure 9: Performance of the multi-site models with the same number of CpG sites as in the real models but randomly selected

The blue bars show the histograms of Pearson correlation coefficients between the actual and predicted baseline eGFR (a-b) and eGFR slope (c-d) of these random models with (a,c) or without (b,d) allowing covariates in the models. The read dashed curves show the fitted normal distributions. The vertical dash lines show the Pearson correlations of the actual models constructed by our procedure. Some random eGFR slope models without allowing covariates had none of the CpG sites with a non-zero coefficient, and thus these models always predicted the same eGFR slope values, leading to a Pearson correlation of 0 with the actual eGFR slopes.

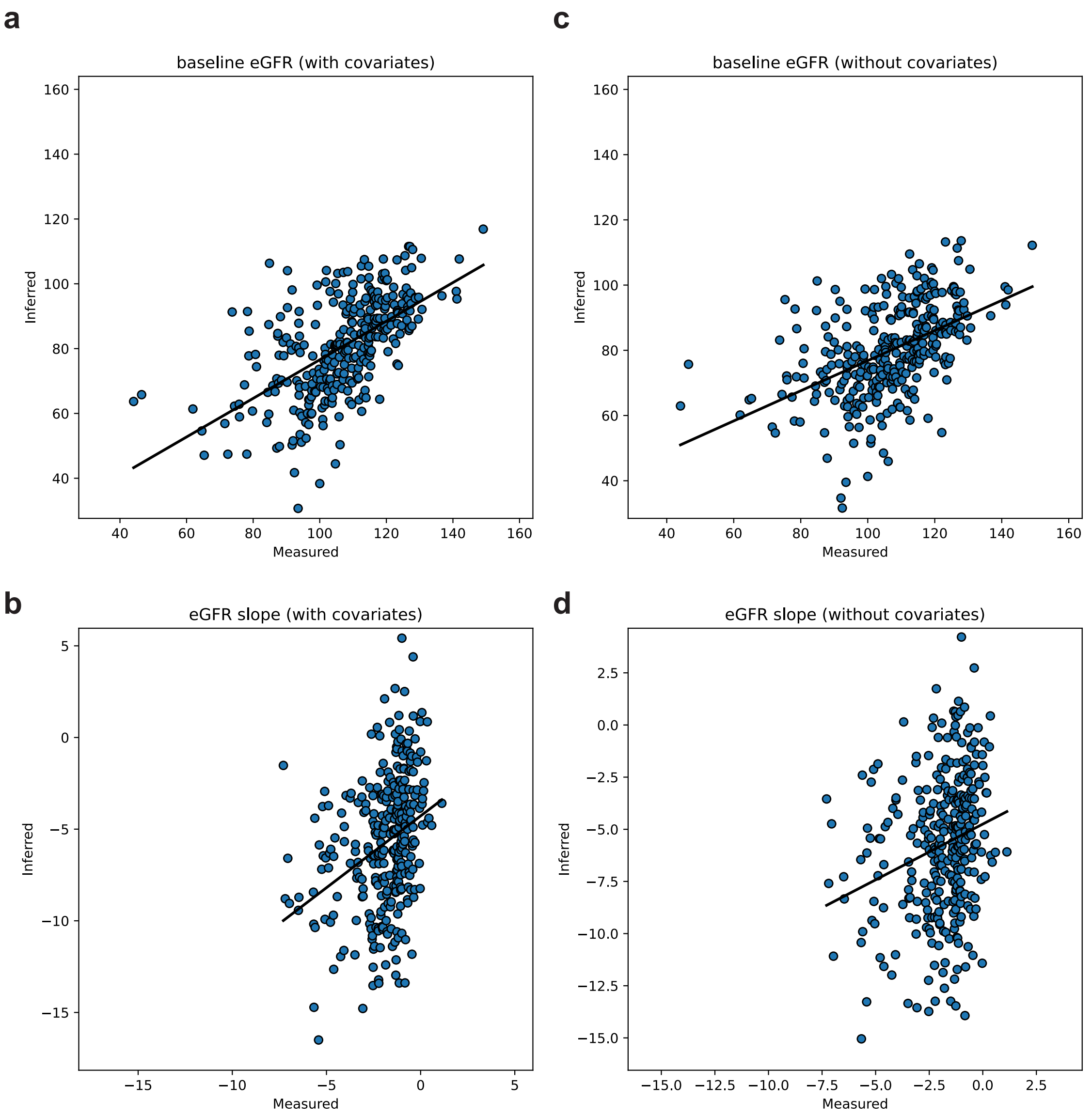

Supplementary Figure 10: Performance of the multi-site models constructed from data of the primary cohort, using CpG sites available to the primary cohort, and applied to the Native American cohort, for baseline eGFR and eGFR slope with covariates in the inputs (a-b) or without (c-d)

Scatter plots of inferred baseline eGFR and eGFR slope against their corresponding actual measurements using selected CpG sites based on the models constructed from the primary cohort and applied to the Native American cohort. In each panel, the black lines mark the best fit lines of linear regression. Source data are provided as a Source Data file.

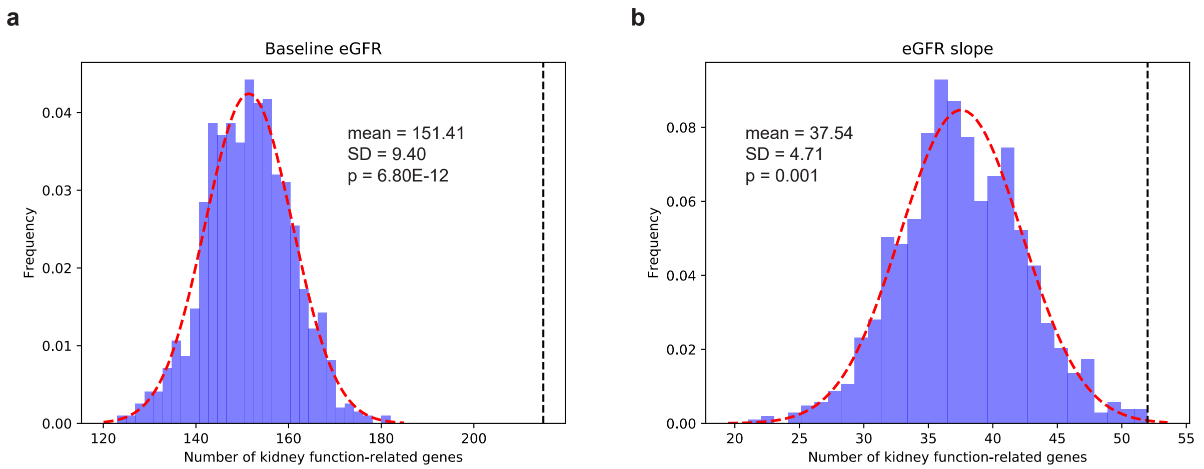

Supplementary Figure 11: Random CpG sites near genes related to kidney function

Comparing the number of genes related to kidney functions that are near random sets of CpG sites and CpG sites identified by our single-site and multi-site analyses, for baseline eGFR (a) or eGFR slope (b). In each panel, the histogram shows distribution of kidney function gene numbers for the random sets of CpG sites (with a size matching the actual number of genes near our identified CpG sites). The red curve shows the fitting Gaussian distribution. The black vertical dotted line shows the actual number of kidney function-related genes near our identified CpG sites. P-value was defined as the total probability of the right tail of the fitted Gaussian distribution beyond the black vertical dotted line.

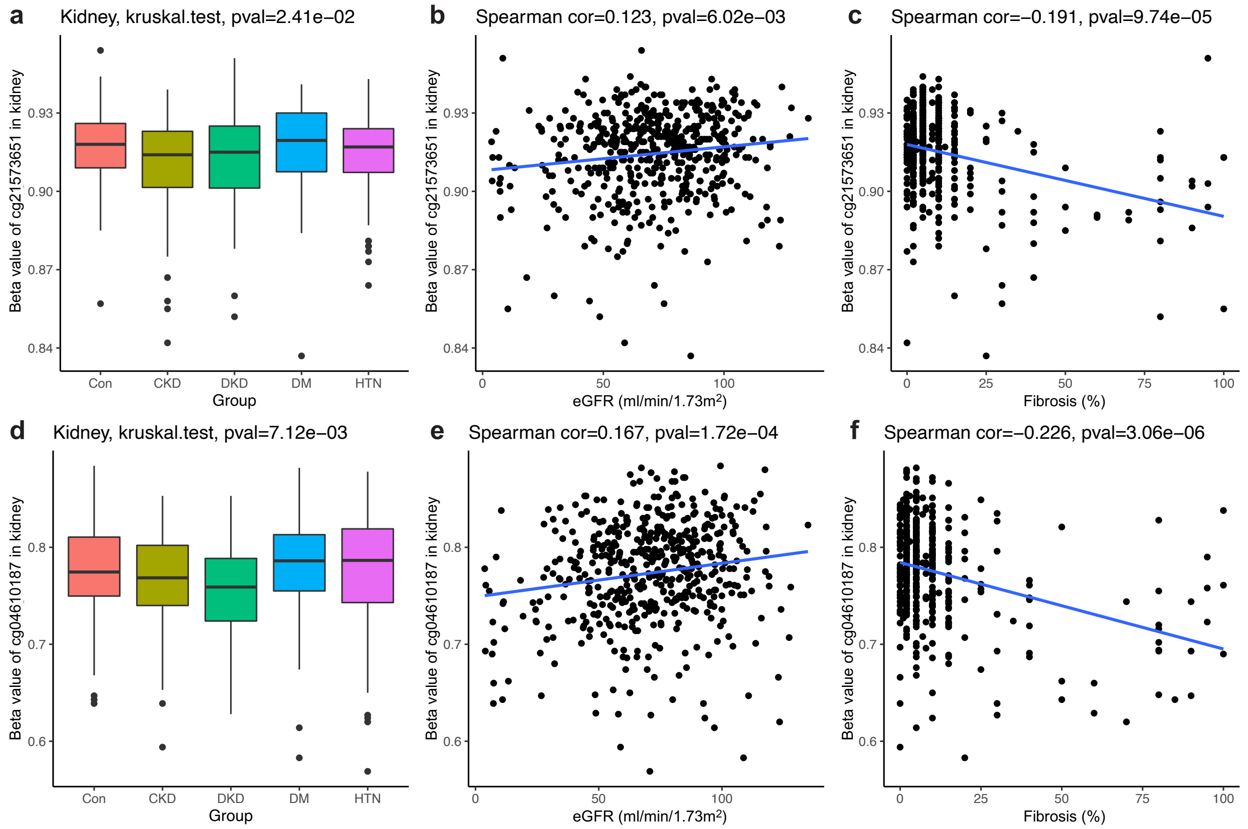

Supplementary Figure 12: Functional Significance of our selected CpG sites' methylation levels in kidney

Methylation levels of cg21573651 (a-c) and cg04610187 (d-e) in kidney samples are significantly different between kidney disease (CKD/DKD) patients and control groups (a,d). They also correlate significantly with eGFR (b,e) and fibrosis (c,f). P-values were computed using two-sided test based on asymptotic *t* approximation. For boxplots in panels (a) and (d), the center lines show the median, and the box limits show the first (Q1) and third (Q3) quartiles (25% and 75%, respectively). Whiskers represents data within the range of Q1-1.5×IQR ~ Q3+1.5×IQR. Outliers beyond the whiskers are shown as points. Con: healthy control, n=113. CKD: eGFR < 60 ml/min/1.73m^2, n=101. DKD: having both CKD and diabetes, n=63. DM: having diabetes but not CKD, n=97. HTN: having hypertension but not CKD, n=132.

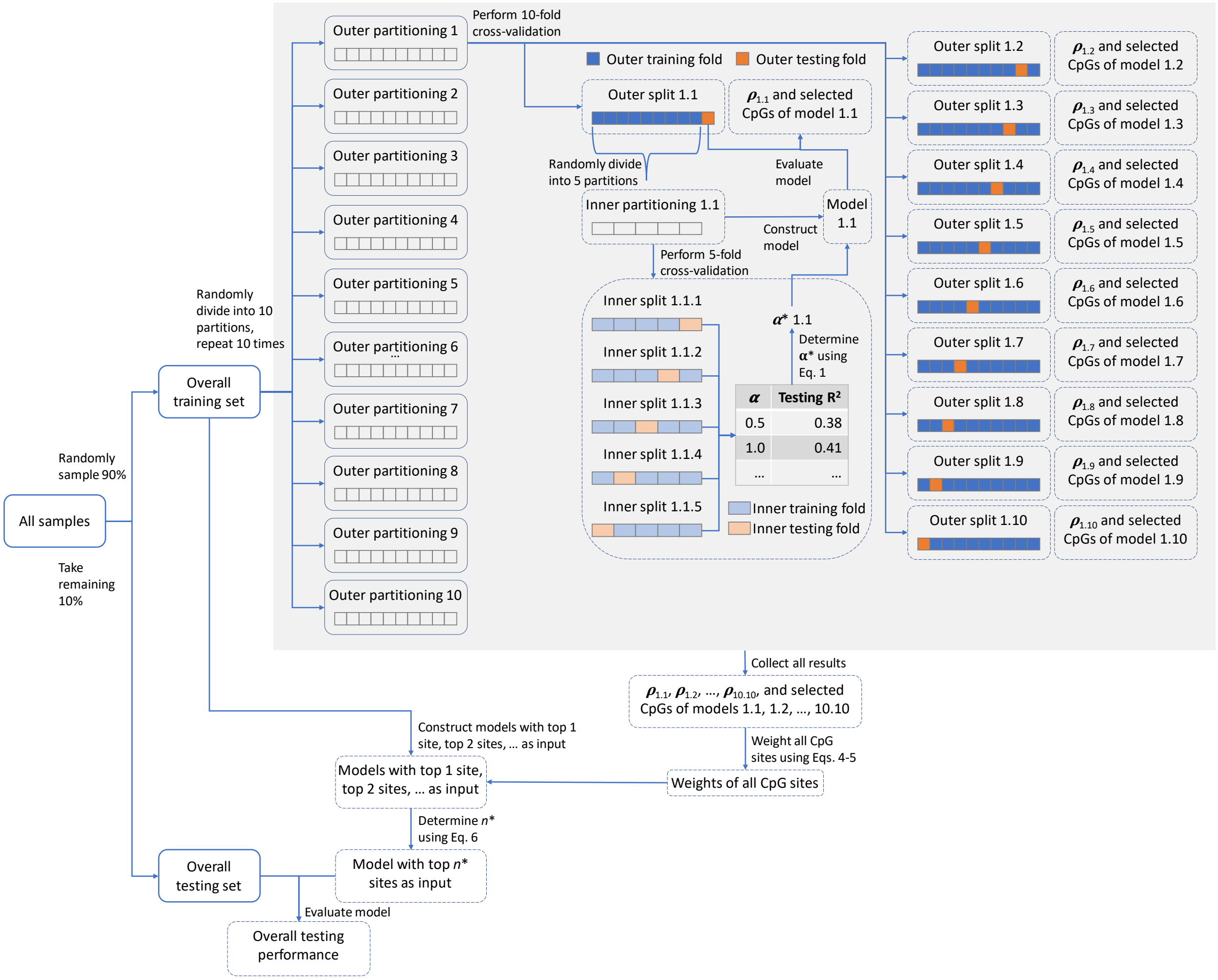

Supplementary Figure 13: Training, parameter tuning and evaluation procedures of the multi-site model

All samples are split into an overall training set (90%) and an overall testing set (10%). The training set is used to assign weights to each CpG site using a 10-fold cross-validation procedure repeated for 10 times. Models are then trained using all samples in the overall training set as examples and different numbers of highest-weight CpG sites as features. The best model is selected using a BIC criterion. It is then applied to the samples in the overall testing set to evaluate model performance. A final model is also constructed using the same procedure but with all 100% samples assigned to the overall training set. This model is evaluated using data from the Native American cohort.

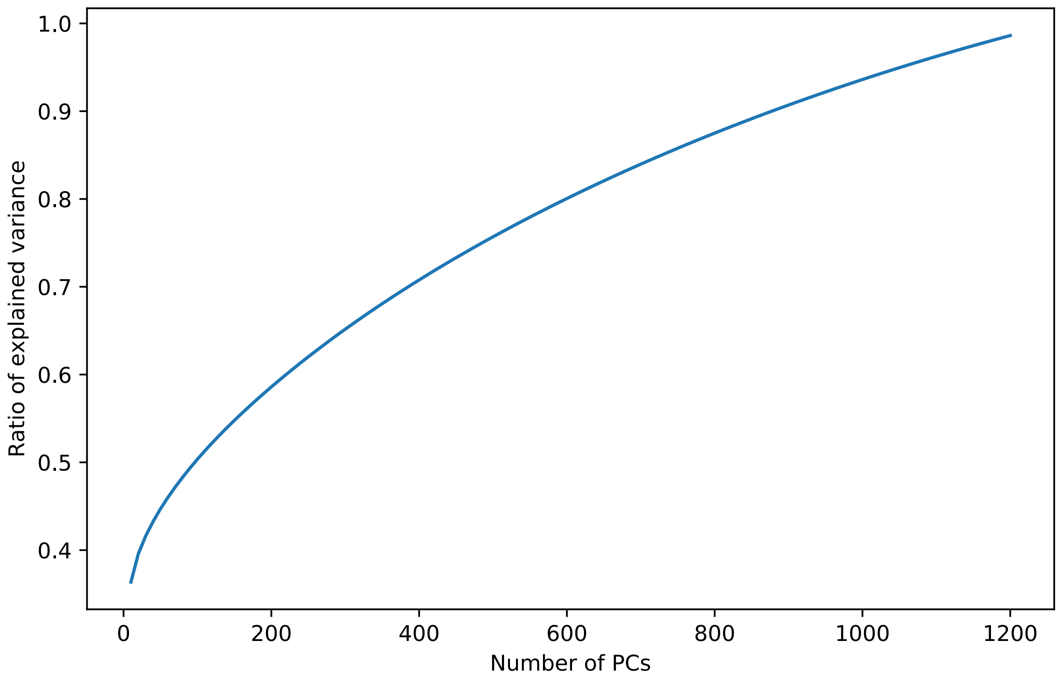

Supplementary Figure 14: Cumulative variance explained by the top PCs of the methylation data

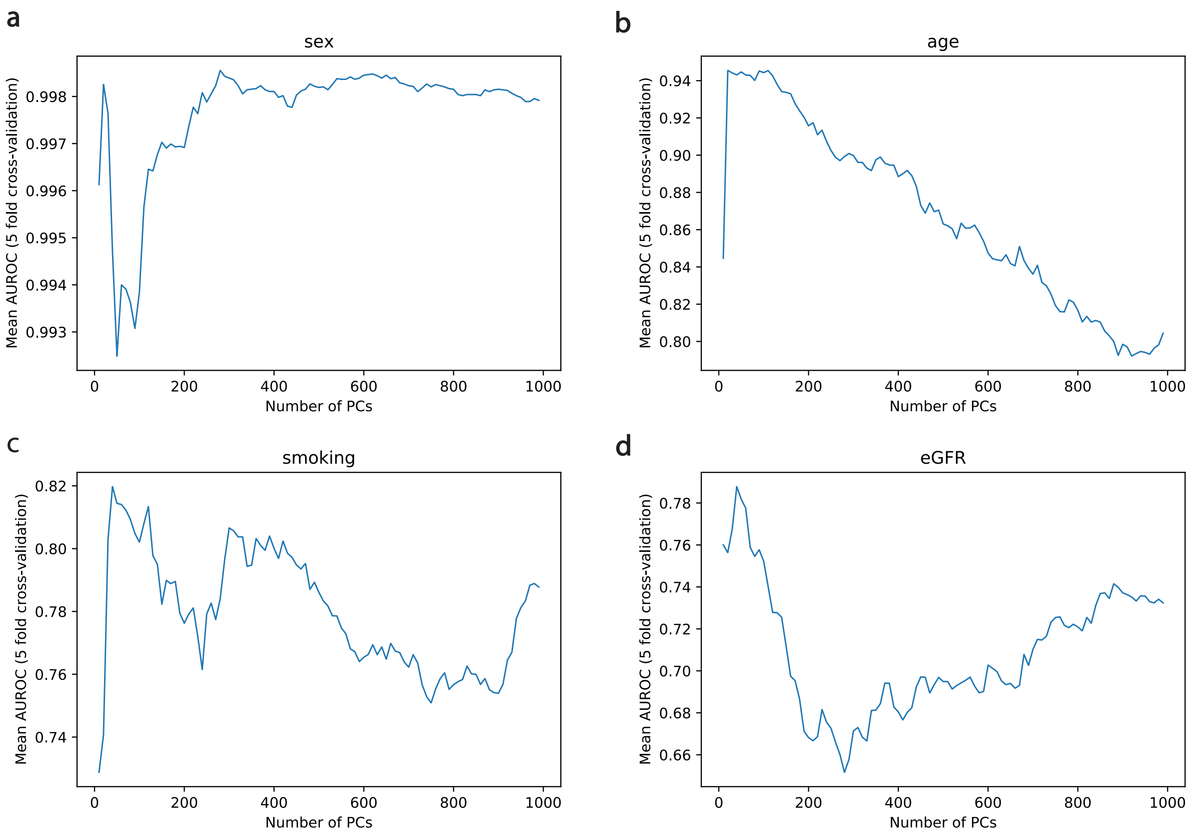

Supplementary Figure 15: AUROC values of the regularized logistic regression models for the four clinical variables most associated with DNA methylation at different number of PCs, including sex (a), age (b), smoking (c), and eGFR (d)

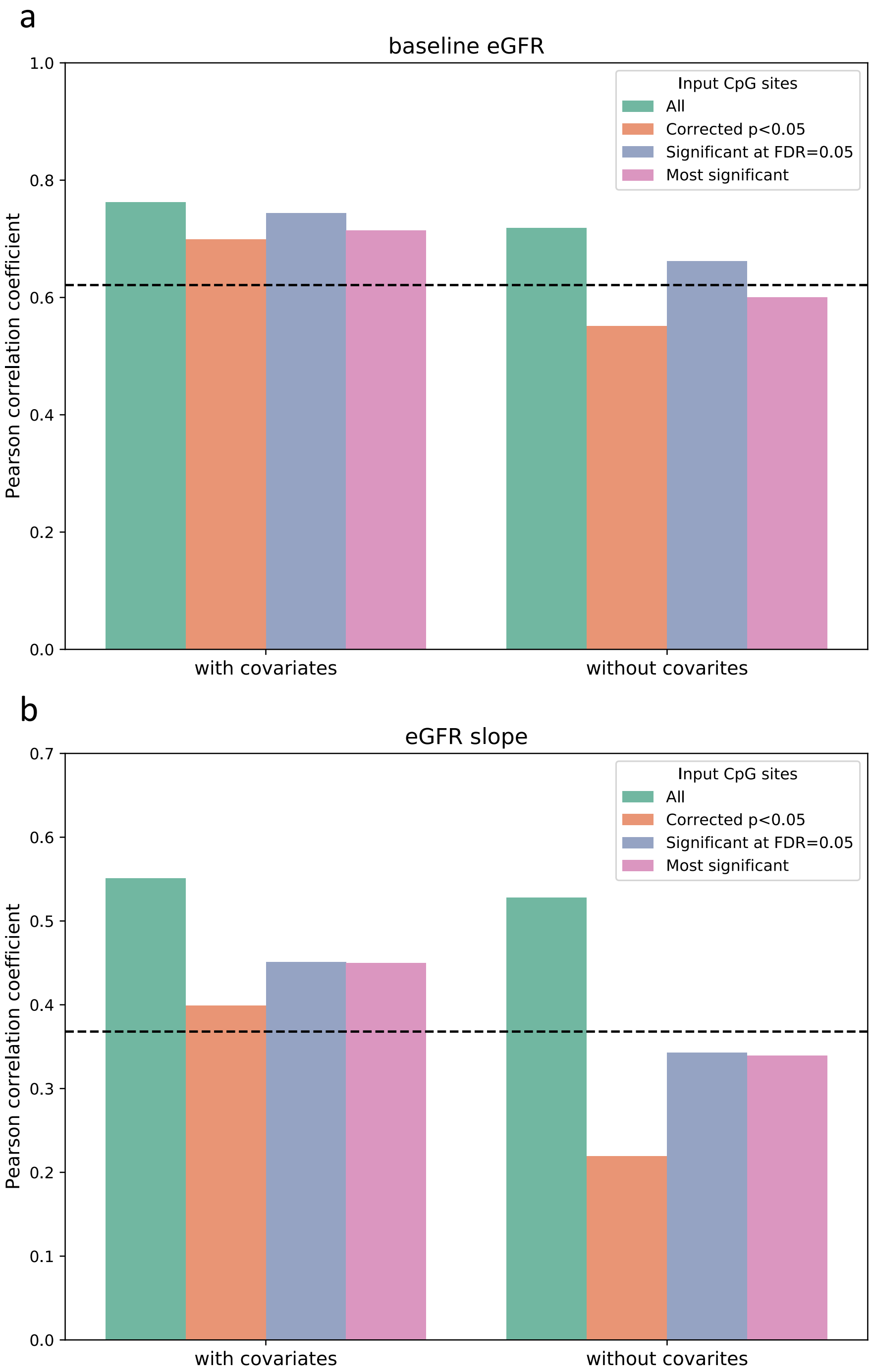

Supplementary Figure 16: Comparing the multi-site models with alternative models

Comparison of the baseline eGFR (a) and eGFR slope (b) multi-site models with alternative models that involve either only CpG sites with Bonferroni-corrected single-site p-values < 0.05, only CpG sites statistically significant at FDR=0.05 in the single-site analysis, or only the set of CpG sites with most significant single-site p-values, with the set size equals the number of sites selected in the final multi-site model. The results are based on 5-fold cross-validation and the horizontal dash lines show the Pearson correlations of models with only covariates as input. Source data are provided as a Source Data file.
