# Supplementary material for: DNA methylation markers for kidney function and progression of diabetic kidney disease
Source: Nat Commun. 2023 May 15;14:2543. doi: 10.1038/s41467-023-37837-7 (PMC10185566; doi:10.1038/s41467-023-37837-7)
Supplement: Supplementary file 2 — Description of Additional Supplementary Files [file 41467_2023_37837_MOESM2_ESM.pdf]

Description of Additional Supplementary Files

Supplementary Data 1: CpG sites significantly associated with baseline eGFR or eGFR slope at FDR=0.05 in the single-site analysis (related to Figure 1, Table 1)

Supplementary Data 2: Meta data of seven previous studies of associating DNA methylation with renal function

Supplementary Data 3: CpG sites included in the final multi-site models of baseline eGFR and eGFR slope learned from all samples of the primary cohort (related to Table 2, 3)

Supplementary Data 4: Support for the functional significance of genes near the CpG sites identified in our single-site and multi-site analyses (related to Figure 3)
