# Supplementary material for: DNA methylation markers for kidney function and progression of diabetic kidney disease
Source: Nat Commun. 2023 May 15;14:2543. doi: 10.1038/s41467-023-37837-7 (PMC10185566; doi:10.1038/s41467-023-37837-7)
Supplement: Supplementary file 7 — Reporting Summary [file 41467_2023_37837_MOESM7_ESM.pdf]

nature portfolio

Corresponding author(s): Ronald C. W. Ma, Kevin Y. Yip

Last updated by author(s): Mar 29, 2023

Reporting Summary

Nature Portfolio wishes to improve the reproducibility of the work that we publish. This form provides structure for consistency and transparency in reporting. For further information on Nature Portfolio policies, see our Editorial Policies and the Editorial Policy Checklist.

Statistics

For all statistical analyses, confirm that the following items are present in the figure legend, table legend, main text, or Methods section.

n/a	Confirmed	
☐	☒	The exact sample size (*n*) for each experimental group/condition, given as a discrete number and unit of measurement
☒	☐	A statement on whether measurements were taken from distinct samples or whether the same sample was measured repeatedly
☐	☒	The statistical test(s) used AND whether they are one- or two-sided<br>*Only common tests should be described solely by name; describe more complex techniques in the Methods section.*
☐	☒	A description of all covariates tested
☐	☒	A description of any assumptions or corrections, such as tests of normality and adjustment for multiple comparisons
☐	☒	A full description of the statistical parameters including central tendency (e.g. means) or other basic estimates (e.g. regression coefficient) AND variation (e.g. standard deviation) or associated estimates of uncertainty (e.g. confidence intervals)
☐	☒	For null hypothesis testing, the test statistic (e.g. *F*, *t*, *r*) with confidence intervals, effect sizes, degrees of freedom and *P* value noted<br>*Give P values as exact values whenever suitable.*
☒	☐	For Bayesian analysis, information on the choice of priors and Markov chain Monte Carlo settings
☒	☐	For hierarchical and complex designs, identification of the appropriate level for tests and full reporting of outcomes
☐	☒	Estimates of effect sizes (e.g. Cohen's *d*, Pearson's *r*), indicating how they were calculated

Our web collection on statistics for biologists contains articles on many of the points above.

Software and code

Policy information about availability of computer code

Data collection	No software was used for data collection.
Data analysis	RnBeads (version 1.6.1), methylumi (version 2.20.0), minfi (versions 1.20.2, 1.28.4), and scikit-learn (version 0.20.3) were used in data analysis.<br><br>Source code for the single-site and multi-site analysis methods produced in this project can be accessed from our GitHub repository, https://github.com/kellyliyichen/eGFR_450k and Zenodo, under GPL-3.0 license.

For manuscripts utilizing custom algorithms or software that are central to the research but not yet described in published literature, software must be made available to editors and reviewers. We strongly encourage code deposition in a community repository (e.g. GitHub). See the Nature Portfolio guidelines for submitting code & software for further information.

Data

Policy information about availability of data

All manuscripts must include a data availability statement. This statement should provide the following information, where applicable:

- Accession codes, unique identifiers, or web links for publicly available datasets
- A description of any restrictions on data availability
- For clinical datasets or third party data, please ensure that the statement adheres to our policy

We have used the human reference genome hg19 in the data analysis.

Individual-level data are protected and are not available because of ethical restriction, as they were not consented for sharing on a public platform. Summary methylation data are available for analysis by qualified researchers who fulfil criteria for access by providing a copy of the research proposal and analysis plan, proof of ethics approval for the planned methylation analysis, and institutional endorsement of server data security. Readers and colleagues who are interested to obtain further information about the study can contact the Hong Kong Institute of Diabetes and Obesity, The Chinese University of Hong Kong, Hong Kong at hkido@cuhk.edu.hk. The summary statistics of significant CpG sites and the multi-site models generated in this study are provided in the Supplementary Information. We have also created a web-based tool using Shiny app so that readers can use the tool to calculate eGFR and eGFR slope based on methylation data, or perform look-up of association between CpG methylation and eGFR. The tool can be accessed at http://hkdbrmlab.shinyapps.io/DKD_EWAS/. Source data are provided with this paper.

Contact person for the Hong Kong Diabetes Register: Professor Ronald Ma, rcwma@cuhk.edu.hk

Contact person for the Native American cohorts: Dr Rob Hanson, rhanson@phx.niddk.nih.gov

Human research participants

Policy information about studies involving human research participants and Sex and Gender in Research.

Reporting on sex and gender	Findings of the study are applicable to both male and female subjects. To eliminate sex-specific biases in our findings, we have included sex as a covariate of our statistical models. Analyses have therefore considered the effects of sex differences. Analyses were conducted using self-reported status of male/female for the subjects. Individual-level data were not suitable / available for sharing.
Population characteristics	The population characteristics of the study population are summarized in supplementary table 1. Among the patients, 19.7% had DKD at baseline, defined as eGFR<60ml/min/1.73m2 (Supplementary Table 1, Supplementary Figure 1). During a median follow-up period of 14.6 (Q1-Q3: 8.3-19.4) years, 33% developed ESKD. During the follow-up period, the included subjects had a median number of eGFR measurements of 29 (Q1-Q3: 15-46), and the median eGFR slope during follow-up was -2.27% (Q1-Q3: -9.11 to -0.65) change of eGFR per year.
Recruitment	Participants were recruited into the Hong Kong Diabetes Register via our diabetes mellitus and endocrine centre, referral from primary care clinics, as well as patients under care at the medical and diabetes clinics of our hospital, the Prince of Wales Hospital.
Ethics oversight	Clinical research ethics approval was obtained from the Joint Chinese University of Hong Kong-New Territories East Cluster Clinical Research Ethics Committee.

Note that full information on the approval of the study protocol must also be provided in the manuscript.

Field-specific reporting

Please select the one below that is the best fit for your research. If you are not sure, read the appropriate sections before making your selection.

[x] Life sciences [ ] Behavioural & social sciences [ ] Ecological, evolutionary & environmental sciences

For a reference copy of the document with all sections, see nature.com/documents/nr-reporting-summary-flat.pdf

Life sciences study design

All studies must disclose on these points even when the disclosure is negative.

Sample size	The epigenome-wide association study was planned so that it would include 400 T2D patients with incident renal complications, matched by 400 control T2D subjects free of complications despite a long disease duration (>10 years T2D). An additional 400 T2D with incident CVD were included as another comparison group, with matched controls. Given some overlap between the control samples, approximately 600 controls subjects with T2D and free of complications were included in total. This study design will provide approximately 100% power at a significance level of α= 10-6, or 98% power at a significance level of α= 10-8 to detect a methylation variable position (MVP) with methylation odds ratio of 1.78, or a difference in mean methylation rate between cases and controls of 10.8%, as calculated in a review article with discussion on different sample size for EWAS (Rakyan VK, Down TA, Balding DJ, Beck S (2011) Epigenome-wide association studies for common human diseases. Nat Rev Genet 12: 529-541.)
Data exclusions	Subjects with methylation data that did not pass the quality control were excluded.
Replication	Findings from the study were replicated in an independent cohort of Native American patients with type 2 diabetes with longitudinal follow-up of renal function and in whom methylation data generated on the same Illumina Infinium array platform is available. The prediction of ESRD using methylation multisite model was replicated using an additional cohort of Native Americans with type 2 diabetes with longitudinal follow-up. In addition, functional analysis in cohorts with methylation data and renal histological changes were examined.
Randomization	Not applicable as the study was an observational study and not a clinical trial.
Blinding	Not applicable as this was not a randomized clinical trial. Methylation data was first evaluated and QC completed before any clinical data was

made available for analysis.

Reporting for specific materials, systems and methods

We require information from authors about some types of materials, experimental systems and methods used in many studies. Here, indicate whether each material, system or method listed is relevant to your study. If you are not sure if a list item applies to your research, read the appropriate section before selecting a response.

Materials & experimental systems

n/a	Involved in the study
☒	☐ Antibodies
☒	☐ Eukaryotic cell lines
☒	☐ Palaeontology and archaeology
☒	☐ Animals and other organisms
☒	☐ Clinical data
☒	☐ Dual use research of concern

Methods

n/a	Involved in the study
☒	☐ ChIP-seq
☒	☐ Flow cytometry
☒	☐ MRI-based neuroimaging
